# The Lineage Differentiation and Dynamic Heterogeneity of Thymic Epithelial Cells During Thymus Organogenesis

**DOI:** 10.3389/fimmu.2022.805451

**Published:** 2022-02-22

**Authors:** Hanchao Gao, Mengtao Cao, Kai Deng, Yang Yang, Jinqi Song, Ming Ni, Chuntao Xie, Wenna Fan, Chunpei Ou, Dinggen Huang, Lizhong Lin, Lixia Liu, Yangyang Li, Huimin Sun, Xinyu Cheng, Jinmei Wu, Cuilan Xia, Xuefeng Deng, Lisha Mou, Pengfei Chen

**Affiliations:** ^1^ Department of Medical Laboratory, Shenzhen Longhua District Central Hospital, Affiliated Central Hospital of Shenzhen Longhua District, Guangdong Medical University, Shenzhen, China; ^2^ Department of Traumatic Orthopedics, Shenzhen Longhua District Central Hospital, Affiliated Central Hospital of Shenzhen Longhua District, Guangdong Medical University, Shenzhen, China; ^3^ Shenzhen Xenotransplantation Medical Engineering Research and Development Center, Institute of Translational Medicine, Shenzhen University Health Science Center, Shenzhen University School of Medicine, First Affiliated Hospital of Shenzhen University, Shenzhen Second People’s Hospital, Shenzhen, China

**Keywords:** thymic epithelial cells, dynamic heterogeneity, thymus organogenesis, cell differentiation, mTEC, cTEC

## Abstract

Although much progress has been made recently in revealing the heterogeneity of the thymic stromal components, the molecular programs of cell lineage divergency and temporal dynamics of thymic epithelial cell (TEC) development are largely elusive. Here, we constructed a single-cell transcriptional landscape of non-hematopoietic cells from mouse thymus spanning embryonic to adult stages, producing transcriptomes of 30,959 TECs. We resolved the transcriptional heterogeneity of developing TECs and highlighted the molecular nature of early TEC lineage determination and cortico-medullary thymic epithelial cell lineage divergency. We further characterized the differentiation dynamics of TECs by clarification of molecularly distinct cell states in the thymus developing trajectory. We also identified a population of Bpifa1^+^ Plet1^+^ mTECs that was preserved during thymus organogenesis and highly expressed tissue-resident adult stem cell markers. Finally, we highlighted the expression of Aire-dependent tissue-restricted antigens mainly in Aire^+^ Csn2^+^ mTECs and Spink5^+^ Dmkn^+^ mTECs in postnatal thymus. Overall, our data provided a comprehensive characterization of cell lineage differentiation, maturation, and temporal dynamics of thymic epithelial cells during thymus organogenesis.

## Introduction

The thymus, a primary lymphoid organ, forms a complex three-dimensional meshwork structure that provides the microenvironment to drive the differentiation, proliferation, and selection of T lymphocytes (1–5). Thymic epithelial cells (TEC), including the functionally and histologically distinct cortical thymic epithelial cells (cTEC) and medullary thymic epithelial cells (mTEC), constitute the main stromal components of thymus. Within this stromal population, cTECs express chemokines such as *Ccl25*, *Cxcl12*, *Il7*, and MHC molecules to initiate lymphoid progenitors seeding, differentiation, and positive selection of immature developing T lymphocytes (6–10). mTECs, which are characterized by promiscuously expressed tissue-restricted antigens, mediate the deletion of self-reactive T cells, thus generating a diverse and self-compatible T-cell repertoire (5, 6, 11).

Thymic rudiment arises from the third pharyngeal pouch region in the mouse embryo at embryonic day 10.5 of gestation (E10.5) with specific expression of *Foxn1* transcription factor (12–15). Transplantation studies in both birds and mice showed that cTECs and mTECs all originated from endodermal cells (14). Then, researchers further demonstrated the existence of thymic epithelial progenitor cells (TEPCs) that contribute to the differentiation of both cTECs and mTECs. By injection of an individual Epcam-positive precursor cell isolated from eYFP-expressing E12.5 thymic rudiments into intact, age-matched wild-type host thymus, the single precursor cell can produce both mTECs and cTECs after 4 weeks of *in vivo* growth (16). Consistently, a different approach using genetic *in situ* labeling in *hK14*: Cre-ERT2;*Rosa26R*-eYFP mice traced the thymic epithelial progenitor cell activities. Cre recombinase under the control of the human *Keratin 14* promoter randomly and rarely switched on the expression of eYFP in thymic epithelial progenitor cells and the eYFP-expressing descendants were observed in both mTEC clusters and cTEC clusters in adult mice (17). Furthermore, reverting a nonfunctional *Foxn1* allele to a functional one in a single postnatal TEPC reawakened its development into a neo-thymi tissue with normal medullary and cortical organization (17). Collectively, these results clearly demonstrated the existence of TEPC in embryonic (at E12.5, Epcam^+^) (16) and postnatal (K14^+^) (17) thymus that produced both mTECs and cTECs. Characterization of the TEPCs has been intensively studied in the recent two decades. The cell surface protein of *Plet1* gene, which is recognized by antibody MTS24 (18), defines a subset of TEPCs in E12.5 (Plet1^+^) (16, 19) or E15.5 (MHCII^+^ Plet1^+^) (20) thymic rudiments. Another study showed the uniform expression of *Plet1* in thymic epithelial cells at E12.5, and that both Epcam^+^ Plet1^+^ and Epcam^+^ Plet1^−^ epithelial cells at E14.5 or E16.5 had similar potential in forming a functional thymus using larger cell numbers in reaggregate fetal thymus organ culture (RFTOC) model (21). Although the differentiation capacity of TEPC was clarified, the differentiation model of TEPC to generate cTECs and mTECs is still elusive.

Besides Plet1, a cTEC marker CD205 was used to identify a subpopulation of CD205^+^ CD40^-^ TECs, which generated functionally competent cortical and medullary micro-environments (22). In addition, a population of IL7^+^ TECs within a subset of CD205^+^ Ly51^+^ CD40^low^ TECs can generate mature CD80^+^ mTECs (23). Using 3xtg^β5t^ transgenic mice in which doxycycline drives the expression of *Cre* recombinase to transcribe the fluorescent protein ZsGreen in β5t-expressing TECs and their progenies, two studies detected both cTECs and mTECs derived from embryonic β5t-expressing precursors (24, 25). These findings suggested a serial progression model of embryonic TEC development in which bipotent progenitors passed through a phase when they expressed cTEC hallmarks prior to generating mTECs (26). In addition, using lineage tracing model β5t-Cre: Rosa26^flox-stop-flox-zsGreen^, Jeanette Baran-Gale and colleagues revealed that the intertypical TECs (Ccl21a^+^ Krt5^+^) arising from β5t^+^ TEC progenitor cells were lineage committed precursors for mature mTECs in adult (27).

In adult thymus, however, the characterization of TEPCs is controversial among studies, and no consensus is reached. For example, Wong et al. identified a rare subset of adult Epcam^+^ MHCII^lo^ UEA-1^-^ a6^hi^ Sca-1^hi^ TECs that generated mature cortical and medullary lineages within fetal thymus reaggregate grafts (28), and Ulyanchenko et al. showed that adult Epcam^+^ MHCII^hi^ UEA-1^-^ Ly51^+^ Plet1^+^ TECs were able to generate both cTEC and mTEC *in vivo* (29). In the postnatal thymus, a population of Epcam^+^ MHCII^-^ Foxn1^lo^ cTECs show sustained colony-forming capacity and can give rise to cTEC and mTEC *in vivo* (30). This study indicated that cortical epithelium contains a reservoir of epithelial progenitors in the postnatal thymus (30). Using transgenic label-retaining cell (LRC) assay, Maude Dumont-Lagacé and colleagues identified a population of LRCs almost exclusively among cTECs expressing high levels of Bmi1, Foxn1, Trp63, and Wnt4, indicating that these cells might be progenitors responsible for TEC maintenance in the adult thymus (31). The differentiation of mature TECs was temporally controlled and stringently associated with developing thymocytes across the lifespan (32). As such, although excellent lines of evidence support the notion that TEPCs in embryonic and adult thymus can generate both cTECs and mTECs, we are still in urgent need of definitive characterization of these cells.

Recently, single-cell transcriptomic analyses have revealed high heterogeneity of medullary thymic epithelial cells including the identification of specific differentiation state of mTECs and rare thymic epithelial subpopulations. Specifically, mTEC in adult thymus are then classified into several subtypes including (1) mTEC I (33), putative TEC progenitors with high expression of *Itga6* and *Ly6a*; (2) mTEC II (33, 34), mature mTECs characterized by high expression of *Aire* and *Fezf2*; (3) mTEC III (33), post-Aire mTECs with expression of *Spink5* and *Krt10*; (4) mTEC IV (33, 35, 36), thymic tuft cells characterized by high expression of *Dclk1* and *Avil* (33, 37); (5) jTEC precursors (38), characterized by the expression of *Pdpn*; (6) pre-AIRE mTEC (35), with high expression of *Ccl21a*; (7) proliferating Aire-expressing mTEC (35); (8) corneocyte-like mTECs (39), with high expression of *Krt1* and *Ivl*; and (9) neuroendocrine, muscle-like myoid, and myelin^+^ TEC subsets in human thymus (36, 39). Despite these fascinating discoveries, the dynamic heterogeneity of TECs during thymus organogenesis is by far largely neglected due to the rarity of TEC populations within total thymic cellularity (33, 34, 36, 40). In particular, the molecular nature of embryonic and adult TEPCs requires further investigation.

Here, we applied scRNA-seq to generate a comprehensive transcriptomic profile of non-hematopoietic cells (enriched for TECs) purified from embryonic to adult stages of thymus. By combination of the scRNA-seq study with *in situ* spatial localization, we reconstructed the temporal dynamics of TEC differentiation.

## Materials and Methods

### Reagents and Materials

**Table:** 

Reagent	Source	Identifier (Cat#)
**Antibodies**		
PerCP Cy5.5 anti-mouse Cd45	BioLegend	147706
Anti-mouse Aqp5	Santa Cruz	sc-514022
Anti Ascl1	Abcam	ab74065
Anti Cdx1	Abclonal	A5712
Anti Hnf1a	Abclonal	A3092
Anti-mouse Aire	eBioscience	14-5934-82
AF488 anti-mouse Ki67	BioLegend	652418
Anti-mouse Ccl6	Abcam	Ab275025
Anti-mouse Emp2	Abcam	Ab174699
Anti-mouse Bpifa1	Solarbio	K005949p
AF488 anti-Krt5	Abcam	Ab193894
Anti-mouse Krt8	BioLegend	904804
Anti-mouse Il4i1	Abclonal	A8378
Anti-mouse Cd16/32	eBioscience	14-0161-85
PE Anti-Rat IgG	BioLegend	407508
PE Anti-Rabbit IgG	BioLegend	406421
		
**Chemicals**		
Probe-Mm-Sox2-C2	Advanced Cell Diagnostic	401041-C2
Probe-Mm-Foxn1	Advanced Cell Diagnostic	482021
Probe-Mm-Ccl21a-C2	Advanced Cell Diagnostic	489921-C2
Probe-Mm-Plet1-C3	Advanced Cell Diagnostic	557941-C3
Probe-Mm-Bpifa1	Advanced Cell Diagnostic	512591
Multiplex Fluorescent Reagent	Advanced Cell Diagnostic	323100
Opal 690 Reagent	Advanced Cell Diagnostic	ASOP690
Opal 570 Reagent	Advanced Cell Diagnostic	ASOP570
Opal 520 Reagent	Advanced Cell Diagnostic	ASOP520
Mounting medium with DAPI	Abcam	Ab104139
Liberase TH	Roche	5401151001
DNase I	Roche	10104159001
Bovine Serum Albumin (BSA)	Sigma-Aldrich	B2064
RPMI 1640 medium	GIBCO	C11875500BT
FBS	GIBCO	10099-141
EDTA solution	Macklin	E885215
40-μm Falcon cell strainer	ThermoFisher	08-771-1
ACK lysis buffer	Gibco	A10492-01
Mouse CD45 microbeads	Miltenyi	130-052-301
QuadroMACS Separator	Miltenyi	130-090-976
LD columns	Miltenyi	130-042-901
**Biological samples**		
Mouse thymus	TEC enrichment (*n*)	Resource
E11.5 thymi	30	Foxn1^EGFP^ reporter
E12.5 thymi	30	Foxn1^EGFP^ reporter
E13.5 thymi	23	wild-type C57BL/6
E14.5 thymi	26	wild-type C57BL/6
E15.5 thymi	25	wild-type C57BL/6
E16.5 thymi	26	wild-type C57BL/6
Newborn thymi	14	wild-type C57BL/6
5-week-old thymi	4	wild-type C57BL/6

### Mice

All mice were maintained under specific pathogen-free conditions at the Animal Facility of Central Laboratory, Shenzhen Longhua District Central Hospital. All animals were handled in accordance with the guidelines of the Animal Care and Use Committee of Guangdong Medical University. Wild-type C57BL/6 mice were purchased from Charles River Animal Center (Beijing, China). Foxn1-EGFP knockin model in C57BL/6 background (hereinafter referred to as Foxn1^EGFP^) was created by CRISPR/Cas9-mediated genome engineering. Briefly, the TGA stop codon of the mouse Foxn1 gene (NCBI Reference Sequence: NM_008238.2) was replaced with “T2A-EGFP” cassette. To prevent the binding and re-cutting of the sequence by gRNA after homology-directed repair, three synonymous mutations, S642 (TCA to AGC), L645 (TTG to CTC), and L647 (CTG to TTA), were introduced. The pups were genotyped by PCR (Primer ID F1, R1, and EGFP F) and confirmed by sequencing. Foxn1^EGFP^ thymi displayed enhanced GFP expression under fluorescence microscope beginning at E10.5–E11.5, which facilitated the dissection of early developing thymi. Foxn1^EGFP^ mice developed normally without gross defects in any organs and had normal reproductive ability.

### Isolation of Mouse Thymic Stromal Cells

Thymi at embryonic day 11.5 (E11.5, *n* = 30, from Foxn1^EGFP^ mice) and E12.5 (*n* = 30, from Foxn1^EGFP^ mice), E13.5 (*n* = 23), E14.5 (*n* = 26), E15.5 (*n* = 25), E16.5 (*n* = 26), newborn (*n* = 14), 5-week-old (*n* = 4) wild-type C57BL/6 mice were dissected and placed into cold 1× PBS. Thereinto, E11.5 and E12.5 thymi from Foxn1^EGFP^ mice were micro-dissected under a fluorescence stereomicroscope. Adhering non-thymus tissue was carefully cleared off with sharp tweezers. Thymi were chopped into small pieces and disintegrated with 0.01% (w/v) Liberase TH and 100 U/ml DNase I in RPMI 1640 (41). Cells were then filtered through a 40-μm cell strainer and washed with 5 ml of MACS buffer (1× PBS with 2 mM EDTA and 0.5% BSA), followed by centrifugation at 200*g* for 5 min. Cells (from newborn and 5-week old thymus) were resuspended with 5 ml of ACK lysis buffer, held on ice for 5 min. Wash the cells with 10 ml of MACS buffer twice. Cells were then subjected to MACS negative separation (mouse CD45 microbeads) according to the manufacturer’s instructions to deplete the CD45^+^ lymphocytes. The stromal cells were then suspended at the concentration of 1 × 10^6^ cells/ml in RPMI 1640–10% FBS, held on ice. E11.5 and E12.5 thymic cells after disintegration were suspended at the concentration of 1 × 10^6^ cells/ml in RPMI 1640–10% FBS for single-cell library construction directly without magnetic depletion.

### Single-Cell Library Construction and Sequencing

We performed single-cell RNA-seq of live CD45^-^ thymic cells by MACS negative separation (about 99% purity confirmed by FACS analysis) to enrich epithelial cells. Library preparation was carried out on fresh cells directly after MACS separation using the Chromium Single Cell 3’ V2 Kit (10X Genomics). Briefly, single cells with cell viability >85% (800–1200 cells/μl, about 15,000 cells for each sample aiming to capture 6,000–10,000 valid cells) were loaded on a 10X Genomics Chromium Single-cell ChIP along with the single-cell master mix and single-cell 3’ gel beads to generate single-cell gel bead-in-emulsions (GEMs). After droplet generation, samples were transferred into PCR tubes and reverse transcription was performed using a C1000 Touch Thermal Cycler (Bio-Rad). Then, cDNA recovery, amplification, and library construction were performed with the Chromium Single Cell 3’ V2 Kit (10X Genomics) following the manufacturer’s instructions. Libraries were sequenced on Illumina Hiseq PE150 platform at an average read depth of about 84,000 reads per cell.

### Quality Control of 10X Genomics Single-Cell RNA-Seq

Following the sequencing, we used fastp v0.20.0 to perform basic statistics on the quality of the raw reads to remove low-quality reads and adapters (42). The reads were then aligned to the mm10 mouse reference genome with the default alignment parameters, filtered, and counted using the Cell Ranger 3.1.1 pipeline provided by 10X Genomics. Common quality control measures for scRNA-Seq including UMI (unique molecular identifiers) count, number of detected genes, and percentage of mitochondrial transcripts were calculated using the Seurat R package (v.3.1, https://satijalab.org/seurat/) (43). The filter criteria of cells and genes were determined after reference to previous studies (34, 44). Genes not detected in any cell were removed from subsequent analysis. To filter low-quality cells, we remove cells that (1) express fewer than 500 unique genes, (2) have less than 2,000 or more than 60,000 UMI counts, and (3) have greater than 10% mitochondrial genes of all expressed genes. The numbers of UMI counts and average gene detection in each sample were summarized in **Table S1**. Data were then normalized using a deconvolution strategy implemented in the R package by computing cell-specific size factors to remove cell-specific biases. Then, the logarithmic normalized counts were used for the downstream analysis. The normalized data were scaled in Seurat. Eight individual samples were merged into one dataset including 58,264 valid cells, which were used for downstream analysis. The integrated dataset consisted of 7,369 cells from E11.5, 1,972 from E12.5, 7,302 from E13.5, 7,552 from E14.5, 8,242 from E15.5, 6,805 from E16.5, 12,985 from newborn, and 6,037 from 5-week old thymi.

### Analysis of Single-Cell RNA-Seq Datasets and Identification of Cell Clusters

We performed principal component analysis (PCA) using the Seurat R Package on a matrix composed of cells and gene expression values. The highly variable genes were identified by running the FindAllMarkers() function in Seurat using the Wilcox test (45). Identification of significant clusters was performed using the FindCluster() in the Seurat package. For each dataset, the first round of clustering (resolution 0.6–1.5) identified 3 major cell types and annotated each cell type by known markers including thymic epithelial cells (*Epcam* and *Cd74*), mesenchymal fibroblast (*Col3a1* and *Pdgfra*), and endothelial cells (*Pecam1* and *Cdh5*). These major cell types were further analyzed in a second round of clustering with the same range of parameters, to identify subclusters within each major cell type.

### Visualization

The dimensionality of a single dataset and an integrated dataset was further reduced and visualized using Uniform Manifold Approximation and Projection (UMAP) with the “RunUMAP” function. Hierarchical clustering and heat map were performed for single cells on the basis of log-normalized expression values of significant genes. Heat maps were generated using the heatmap.2 function from the gplots v3 R package with the default complete-linkage clustering algorithm. Log-normalized gene expression values were plotted for each cell as a violin plot with an overlying dot plot in R package.

### Differential Expression Analysis

To identify cluster-enriched or cell type-enriched genes, we performed *wilcox. test* in R to evaluate the significance of each gene. Genes with adjusted *p*-value less than 0.05, false discovery rate (FDR) less than 0.01, at least 0.5 average fold change (log scale), and at least 25% detection (percentage of cells expressing a particular gene in a cluster or cell type) were considered as differentially expressed genes.

### Pseudotime Trajectory Analysis for Thymic Epithelial Cells

Thymic epithelial cell fate decisions and pseudotime trajectories were constructed by the Monocle2 R package (v 2.10.1, http://cole-trapnell-lab.github.io/monocle-release/). A total of 30 to 100 significantly highly expressed genes (FDR < 0.01, logFC > 1) for each subcluster were selected and combined as the set of ordering genes and performed dimension reduction and trajectory analysis. The early developing TECs (E11.5–E14.5) and mTECs (E14.5–adult thymus) were analyzed separately.

### Ligand–Receptor Interaction Analysis

To identify potential regulating relationships between cell types (here, we focused on mesenchymal fibroblast and TECs) within thymic microenvironment, we scored a given ligand–receptor interaction as the product of average ligand expression and the average receptor expression (46). We used a reference list of known, literature-supported ligand–receptor pairs (47) and excluded the genes with no or rare expression in our data (genes excluded with average expression <0.1).

### Immunofluorescence Staining and Microscopy

All immunostainings were performed on thymus sections obtained from C57BL/6 mice at the age indicated. Dissected thymi or whole embryos (for E11.5, E12.5, and E13.5 thymi) were embedded in optimal cutting temperature compound and sectioned in 10-μm sections. To perform immunofluorescent staining, sections were first washed with 1× PBS, followed by fixation in cold acetone for 5 min. Then, the sections were rinsed in PBS for 3 times and permeabilized with 0.3% Triton-X in PBS–1% BSA buffer for 10 min at room temperature. After blockade with 1% BSA in PBS for 30 min at room temperature, the primary antibodies were added in PBS–1% BSA buffer, hatching overnight at 4°C. Sections were washed in PBS–0.2% Tween once and then in PBS 3 times for 5 min at room temperature. Secondary antibody staining was performed at room temperature for 2 h in PBS–1% BSA. Sections were washed in PBS–0.2% Tween once and then in PBS 3 times for 5 min at room temperature. Sections were mounted with mountant (with DAPI) and visualized with a confocal microscope by NIS-Elements software using the 10× and 20× objective. The same settings were applied to all images shown for each experiment. All experiments were replicated at least 2 times, with 2 biological replicates for each experiment.

### Single-Molecule RNA Fluorescence *In Situ* Hybridization

Optimal cutting temperature compound embedded thymuses were sectioned in 10-μm sections and processed as per the RNAscope multiplex fluorescent reagent kit V2 (323100) manual with some modifications. Briefly, fix the slides in cool 4% paraformaldehyde for 15 min, rinse the slides 2 times, dehydrate the slides with 50%, 70%, and 100% ethanol sequentially, air dry for 5 min, incubate the slides with hydrogen peroxide for 10 min at RT, rinse the slides with 1× PBS, incubate the slides with protease III for 8 min at RT, rinse the slides with 1× PBS, incubate the slides with probe mix in a 40°C incubator for 3 h, rinse the slides with wash buffer twice, hybridize AMP1, 2, and 3 successively, develop fluorescein signal for each channel successively, and mount the slides with mountant (with DAPI). Slides were visualized with a confocal microscope by NIS-Elements software using the 10× and 20× objective. The same settings were applied to all images shown for each experiment. All experiments were replicated at least 2 times, with 2 biological replicates for each experiment.

### Statistical Analyses

For the analysis of gene expression in scRNA-seq data, all single-cell sequencing data statistical analysis was performed in R using Seurat. Wilcoxon Rank Sum test was applied for comparisons. *p*-values were adjusted based on Bonferroni correction. Statistical significance was accepted for *p* < 0.05.

## Results

### Non-Hematopoietic Cellular Composition of the Mouse Thymus Across Life

**Figure 1:**
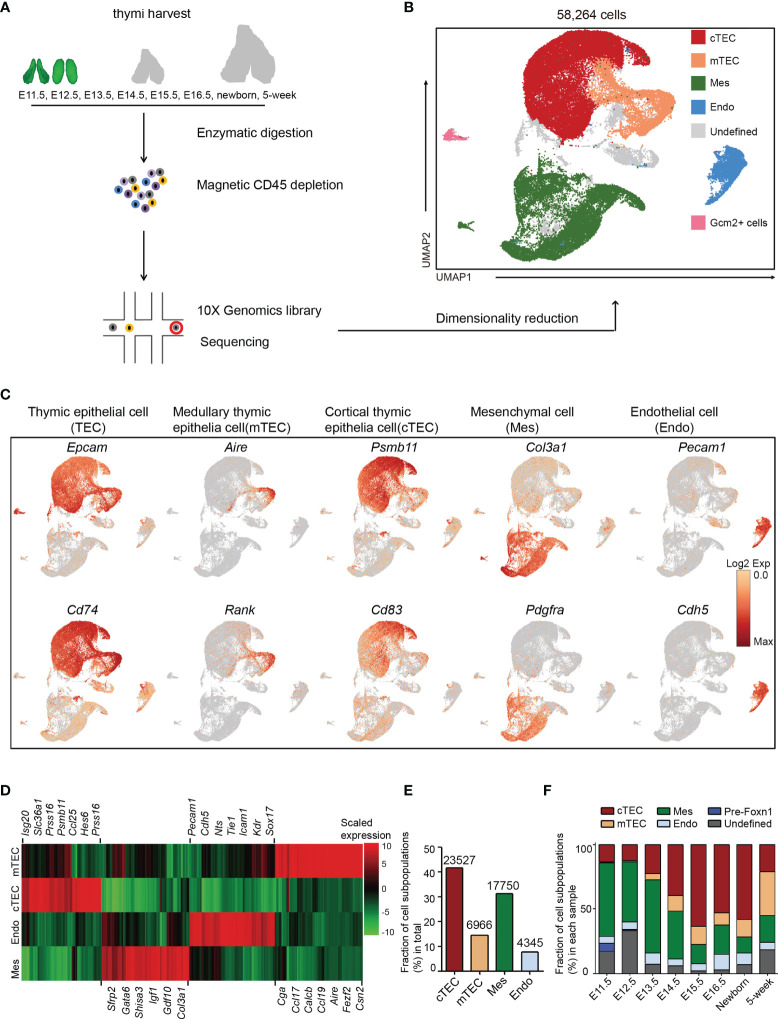
Non-hematopoietic cellular composition of the developing thymus. **(A)** Schematic of single-cell transcriptome profiling of the developing thymus. **(B)** UMAP visualization of the cellular composition of the thymus colored by cell type (cTEC, cortical thymic epithelial cell; mTEC, medullary thymic epithelial cell; Mes, mesenchymal fibroblast; Endo, endothelial cell). **(C)** UMAP visualization of the expression of curated feature genes for cell cluster identification. (See also **Figure S1**) **(D)** Heat map showing differently expressed genes in four major cell types: mTEC, cTEC, Endo, and Mes. Expression levels were maximum-normalized and smoothed. Genes were grouped by their expression patterns. (See also **Table S3**) **(E)** The fraction of major cell types: cTEC, mTEC, Mes, and Endo in our scRNA-seq data. The numbers above indicated the number of cells for each cell type. **(F)** The percentages for each cell type across the development from E11.5 to adult. (See also **Table S2**).

We performed scRNA-seq on non-hematopoietic cells purified from murine thymi at E11.5, E12.5, E13.5, E14.5, E15.5, E16.5, newborn, and 5 weeks old, for 8 developmental stages (**Figure 1A**). To aid with mechanical isolation of the early embryonic thymi, E11.5 and E12.5 thymi were dissected from *Foxn1*-driven enhanced green fluorescent protein (EGFP) reporter mice in which expression of EGFP was fused with Foxn1. Other thymi were dissected from wild-type C57BL/6 mice. TECs are rare in the total thymic cellularity, with the proportion of less than 1% in adult and less than 10% in embryonic thymus except for E11.5 and E12.5 (**Figure S1A**). For the sake of effective enrichment of thymic epithelial cells in scRNA-seq data, we loaded as input the thymic non-hematopoietic cells by magnetic depletion of CD45^+^ cells. After quality control (see *Materials and Methods*), ∼84,000 mean reads per cell and 3,000 median genes per cell could be detected in the transcriptomes of 58,264 single cells, including 30,959 TECs from 8 stages of thymi (**Table S2**).

Following gene expression normalization for read depth and mitochondrial read count, single-cell data were projected into a reduced-dimensional space using UMAP and were clustered based on the top principal components (48) (**Figure 1B**). Three major cell types were readily recognized: thymic epithelial cells (TEC) consisting of cTEC and mTEC, mesenchymal fibroblast cell (Mes), and endothelial cell (Endo). A small population of Gcm2^+^ cells were contaminants of thyroid tissue from embryonic samples (**Figure 1B**). The undefined clusters were mainly developing T cells due to leakage from magnetic depletion (**Figure 1B**). TECs (markers: *Epcam* and *Cd74*), mTECs (markers: *Aire* and *Rank*), cTECs (markers: *Psmb11* and *Cd83*), Mes (markers: *Col3a1* and *Pdgfra*), and Endo (markers: *Pecam1* and *Cdh5*) were annotated by the expression of their feature genes and literature evidence (**Figures 1C, D** and **S1B–S1E** and **Table S3**).

Thymic epithelial cells constituted the main cell types and were well represented in our dataset, including 12% of mTECs and 40% of cTECs (**Figure 1E**). Mesenchymal fibroblasts represented the second largest cell type (30%) and endothelial cells accounted for 7% (**Figure 1E**). Importantly, when comparing between samples, mesenchymal fibroblasts constituted the main thymic cellularity (>50%) before E13.5 and then continuously decreased with age (**Figure 1F**). cTECs were frequently detected as early as E11.5 (13%) and gradually increased in proportion till birth. However, the mTECs were rarely detected at E11.5–E12.5. They began to appear at E13.5 (5%) and gradually exceeded the number of cTECs in adult thymi (**Figure 1F** and **Table S2**). A small population of Foxn1^-^ cells that highly expressed *Sox2* was mainly detected in E11.5 thymi (**Figure 1F** and **Table S2**).

### Molecular Characteristics of TECs at cTEC–mTEC Lineage Divergency

**Figure 2:**
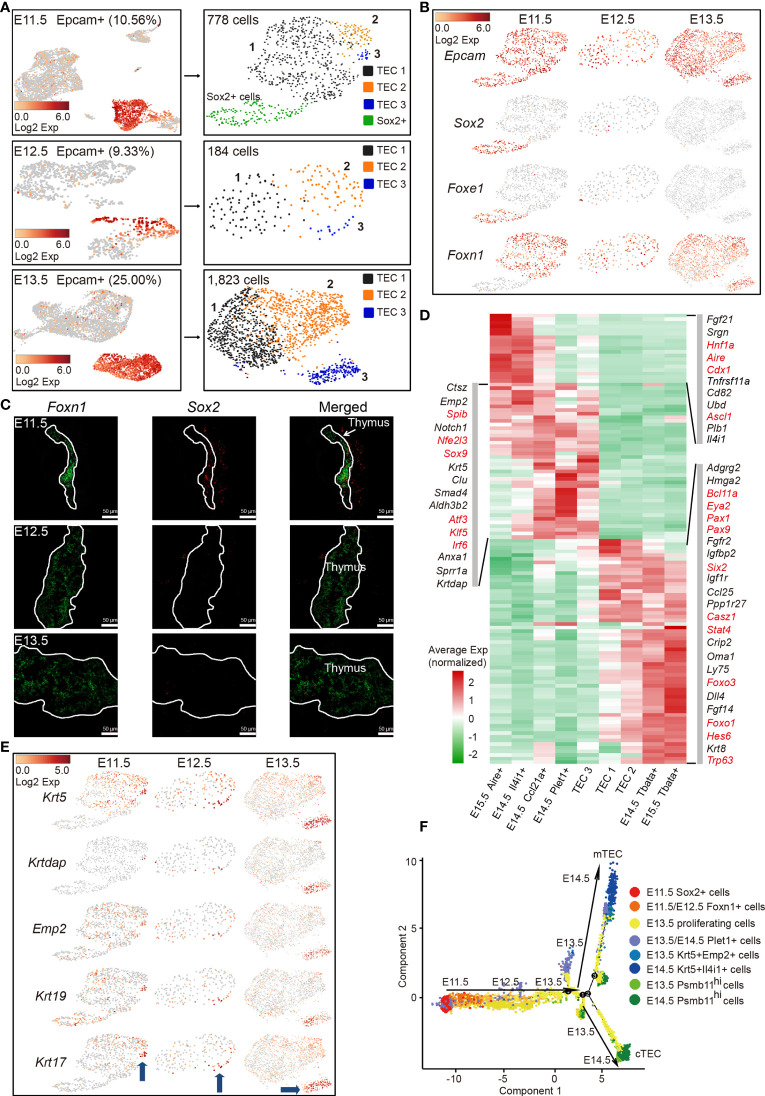
Molecular features of early TECs and cortico-medullary thymic epithelial cell lineage divergency at E11.5–E13.5. **(A)** UMAP visualization of non-hematopoietic cells from E11.5, E12.5, and E13.5 thymus (left panel, numbers indicated the percentages of Epcam^+^ cells). UMAP visualization of Epcam^+^ cells (right panel, numbers indicated the number of Epcam^+^ cells analyzed). **(B)** UMAP visualization of the expression of curated feature genes for cell cluster identification. (See also **Figure S2A**.) **(C)** Single-molecule RNA fluorescence *in situ* hybridization in fetal thymus slides with probes targeting *Foxn1* and *Sox2*. **(D)** Heat map showing gene expression features in clusters of TEC 1, TEC 2, and TEC 3 from E11.5–E13.5 thymus, and mTEC and cTEC from E14.5 and E15.5 thymus. Expression levels were maximum-normalized and smoothed. Genes were grouped by their expression patterns. Genes in red are transcription factors. (See also **Figure S2D**.) **(E)** UMAP visualization of the expression of curated feature genes specific for cell cluster TEC 3; blue arrows indicated the position of TEC 3 in each sample. **(F)** The ordering of Epcam^+^ cells from E11.5–E14.5 thymus along pseudotime in a two-dimensional state space defined by Monocle2. Cell orders were inferred from the expression of most dispersed genes across Epcam^+^ cells. Each point corresponded to a single cell, and each color represented a cell cluster.

Thymic epithelial cell heterogeneity in adult thymus has been revealed by recent studies; however, the molecular characteristics of TECs at the single-cell level in early thymic rudiment are missing (33–39). In particular, the molecular nature at the checkpoint of cortico-medullary thymic epithelial cell divergency needs urgent investigation. To this aim, single-cell transcriptomes of Epcam-expressing epithelial cells from E11.5, E12.5, and E13.5 thymi were analyzed. The increased proportion of Epcam-expressing cells (25%) at E13.5 compared with E11.5 and E12.5 (10%) indicated the rapid expansion of TECs at this developmental stage (**Figure 2A**, left panel). Unsupervised clustering revealed 4 subpopulations (TEC 1-3 and Sox2^+^ cells) from E11.5 and 3 subpopulations (TEC 1–3) from E12.5 or E13.5 thymi, respectively (**Figure 2A**, right panel). A subpopulation of Epcam^+^ Sox2^+^ Foxe1^+^ Foxn1^-^ cells was mainly detected in E11.5 thymi and quickly diminished in samples thereafter (**Figure 2B**). Gene expression profiling distinguished this population from others by high expression of *Sox2*, *Igfbp2*, *Igfbp5*, *Klf5*, *Krt7*, *Cldn6*, *Anxa8*, and *Foxe1* (**Figure S2A**). The expression of *Sox2* was further validated by single-molecule RNA fluorescence *in situ* hybridization. The cells that highly expressed *Sox2* were observed in the vicinity of Foxn1-expressing thymic epithelial cells (**Figure 2C**). Sox2 expression was previously detected in the third pharyngeal pouch in E9.5–E10.5 mouse embryo by *in situ* hybridization (49). Together, based on these results, we speculated that these Epcam^+^ Sox2^+^ Foxe1^+^ Foxn1^-^ cells may serve as a transition before Foxn1^+^ thymic epithelial cell lineage determination. However, lineage tracing experiments are further needed to confirm this possibility.

Specifically, TEC 1, TEC 2, and TEC 3 were characterized by high expression of *Foxn1* (**Figure 2B**), consistent with its first expression in thymic epithelium at E11.5 (12, 50). TECs at E11.5, E12.5, and E13.5 expressed a handful of common TEC marker genes *Epcam*, *Foxn1*, *Krt8*, *Ctsl*, and *Psma7* (**Figure 2B**, and **Figure S2B**). Importantly, early TECs highly expressed mature cTEC-specific genes including transcriptional regulators *Pax1*, *Pax9*, *Six1*, *Eya2*, *Mtf2*, protease *Psmb11*, *Prss16*, lymphocyte chemotactic factors *Ccl25*, *Cxcl12*, *Il-7*, and metabolic regulators *Pltp*, *Comt*, and *Ndufa11* (**Figures S2C, D**). We then compared TEC 1, TEC 2, and TEC 3 with mTECs and cTECs in later developmental stages (E14.5 and E15.5) to investigate the transcriptomic differences among these populations. Hierarchical cluster analysis of differentially expressed genes (DEGs) revealed the upregulation of mTEC-specific transcription factors *Spib*, *Nfe2l3*, *Sox9*, *Atf3*, *Klf5*, and *Irf6* in TEC 3, and cTEC-specific genes *Oma1*, *Ly75*, *Fgf14*, and *Trp63* in TEC 2, indicating that TEC 3 and TEC 2 represented the onset of cortico-medullary thymic epithelial cell divergency at E11.5–E13.5 (**Figures 2D** and **S2E**). It was worth noting that some of the cTEC-specific genes were already highly expressed in TEC 1, but mature mTEC-specific genes were rarely detected in this population (**Figure 2D**). In addition, TEC3 cells upregulated the expression of several keratinocyte differentiation regulators and cell surface protein genes such as *Emp2*, *Krtdap*, *Krt5*, *Krt17*, and *Krt19* (**Figure 2E**).

The complete transcriptome for a large number of early embryonic TECs allowed us to gain insights into the functional states of and relationship among these cells. We ordered cells in a pseudotemporal manner using Monocle 2 algorithm (51) to indicate their developmental trajectories. Cells from each cluster aggregated based on their expression similarities, and cell clusters from E11.5 to E14.5 thymi formed into a relative process in pseudotime that began with the Sox2^+^ cells, followed by Foxn1-expressing cells (mainly from E11.5 to E12.5) before bifurcation (**Figure 2F**). However, TEC 1 from E13.5 that were characterized by high expression of cell cycle-related genes bifurcated into two diverse branches, representing two major cell lineages in the late reprogramming stage. Krt5^+^ cells from E13.5 to E14.5 constituted one terminal while Psmb11^hi^ cells constituted the other terminal (**Figure 2F**).

Together, these results revealed that early thymic epithelial cells initially gained common TEC- and cTEC-specific gene expression signatures, but did not express mTEC-specific genes, indicating that mature mTEC was a more specialized cell type and maturation of cTEC was probable an intrinsic process. TECs from E11.5 to E13.5 were relatively homogenous based on their common gene expression signatures. Thus, our data suggested that E11.5–E13.5 may represent a stage that starts cortico-medullary thymic epithelial cell lineage divergency, consistent with detection of early mTECs at E13.5 (34).

### The Dynamic Heterogeneity of mTEC During Development

**Figure 3:**
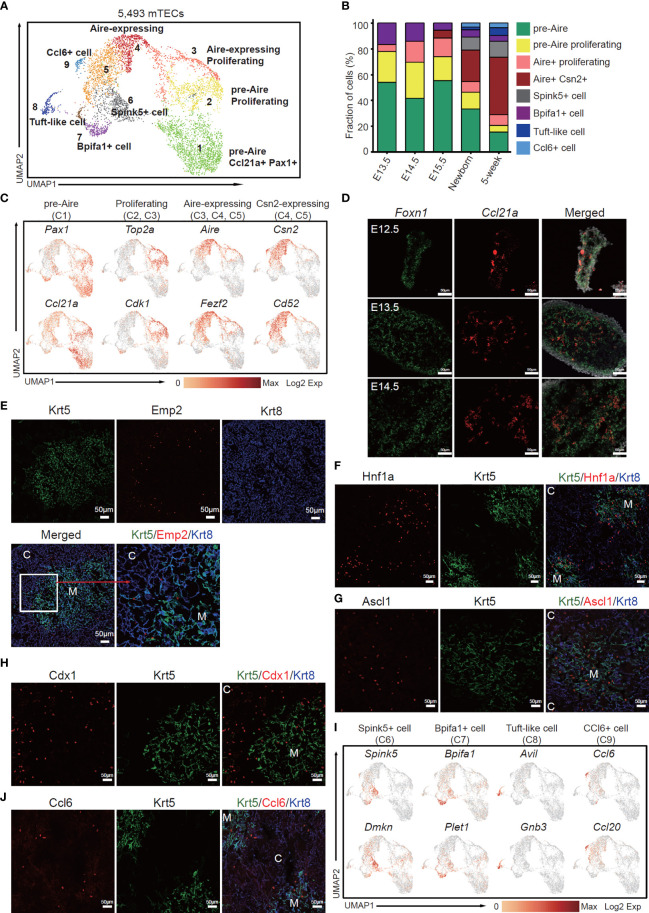
The temporal dynamics of mTEC diversity during thymus organogenesis. **(A)** UMAP visualization of 5,493 mTECs with 9 subclusters. **(B)** Percentages of 8 mTEC subtypes across the development from E13.5 to adult. **(C)** UMAP visualization of the expression of curated feature genes specific for 4 mTEC subtypes. (See also **Figure S3A**.) **(D)** Single-molecule RNA fluorescence *in situ* hybridization in fetal thymus slides with probes targeting *Foxn1* and *Ccl21a*. **(E–H)** Immunofluorescence staining of 5-week thymus slides with antibodies to Krt5, Emp2 **(E)**, Hnf1a **(F)**, Ascl1 **(G)**, Cdx1 **(H)**, and Krt8 (C, cortex; M, medulla). **(I)** UMAP visualization of the expression of curated feature genes specific for 4 mTEC subtypes: Spink5^+^ cells, Bpifa1^+^ cells, tuft-like cells, and Ccl6^+^ cells. (See also **Figure S4A**) **(J)** Immunofluorescence staining of 5-week thymus slides with antibodies to Krt5 (green), Ccl6 (red), and Krt8 (blue). All staining experiments were replicated at least 2 times, with 2 biological replicates for each experiment.

Next, we investigated the dynamics of mTEC across later development from E13.5 to adult. Although the early TECs were relatively homogenous and expressed a large number of cTEC-specific genes, unsupervised clustering of TECs based on gene expression similarities distinguished clusters of mTECs from cTECs, resulting in a total of 5,493 cells with mTEC characteristics from E13.5, E14.5, E15.5, newborn, and adult thymi (**Figure 3A**). Reanalysis of these mTECs defined 9 subclusters, each distributed at a distinct position within the two-dimensional projection (**Figure 3A**), reflecting distinct transcriptional and molecular characteristics. Among these cell types, relative abundance changed drastically during thymus organogenesis. Obviously, the pre-Aire mTECs including mTEC C1 (cluster 1) and mTEC C2 dominated the embryonic mTEC components (70%–80%), and decreased in proportion after birth (**Figure 3B**). The proliferative Aire-expressing mTECs (mTEC C3) were first detected at E13.5 and persisted during development (**Figure 3B**). The mature mTECs expressing *Aire* and *Csn2* (mTEC C4 and C5) were first detected at E15.5 and increasingly accumulated in quantity after birth (**Figure 3B**). The Spink5^+^ cells (mTEC C6), tuft-like cells (mTEC C8), and Ccl6^+^ cells (mTEC C9) first emerged in newborn thymus and expanded in adult (**Figure 3B**). The mTEC C7 represented a population of Bpifa1^+^ Plet1^+^ cells that were preserved across thymus development (**Figure 3B**).

Specifically, mTEC C1 was characterized by high expression of *Pax1*, *Ccl21a* (**Figure 3C**). Expression of *Pax1* was found in a large proportion of thymic epithelial cells at early stages of developments and restrained in cTECs in the adult (**Figure S3A**), as previously reported (52). Most of the embryonic mTECs highly expressed *Ccl21a* and *Emp2*; however, in the adult, expression of *Ccl21a* and *Emp2* was restrained in a subpopulation of Aire-negative cells (**Figure S3A**). Furthermore, single-molecule RNA fluorescence *in situ* hybridization revealed that *Ccl21a* expression started as early as E12.5 before Aire expression (**Figure 3D**). Immunofluorescence staining identified Emp2 expression in TECs resident at the cortico-medullary junction in embryonic and adult thymus (**Figures 3E** and **S3B**). Podoplanin (Pdpn)-expressing TECs located at the cortico-medullary junction were reported as lineage committed progenitors that gave rise to mature mTECs (53). Although *Pdpn* was expressed in both cTEC and mTEC in embryonic and newborn thymus, its expression was restrained in a subpopulation of TECs that also expressed *Emp2* (**Figure S3A**). Thus, these results together indicated that mTEC C1 population may represent an early state of mTEC differentiation.

mTEC C2 and mTEC C3 were characterized by high expression of cell cycle regulating genes such as *Top2a* and *Cdk1* (**Figures 3C** and **S3C**). Among these, mTEC C3 simultaneously expressed autoimmune regulator *Aire* (**Figure 3C**), representing a subpopulation of Aire-expressing amplifying cells, which are further confirmed by *in situ* staining of *Aire* and *Ki67* (**Figure S3D**). mTEC C4 was highlighted with high expression of *Aire*, *Fezf2*, and other known mature mTEC marker genes (**Figures 3C** and **S3C**). mTEC C5 mainly contained cells of postnatal thymus, and highly expressed *Csn2* and *CD52* (**Figures 3C** and **S4A**), which were markers of mature mTECs (33). Hepatocyte nuclear factor 1-alpha (*Hnf1a*), a transcription factor that activates differentiated acinar cell programs (54) in pancreas, was highly expressed in mature mTECs (**Figure 3F**). An achaete-scute homolog 1 (*Ascl1*) that acts as a chromatin remodeling factor to promote neuronal differentiation (55) was also enriched in medulla and some cTECs (**Figure 3G**). Caudal-related homeobox (CDX) transcription factors, *Cdx1* and *Cdx2*, have long been identified as intestine-specific modulators for directing intestinal differentiation and maintenance of the intestinal phenotype (56). Our data revealed high expression of *Cdx1* but not *Cdx2* in mTEC C3, C4, and C5 (**Figure S3C**). Immunofluorescence staining confirmed its expression in medulla and adjacent cTECs (**Figure 3H**). mTEC C6 was characterized by expression of *Spink5* and *Dmkn*, indicating a subpopulation of mTECs with terminal differentiation phenotype (**Figures 3I** and **S4A**) (33). mTEC C7 included cells from all developmental stages (**Figure 3B**), and highly expressed *Bpifa1* and *Plet1* (**Figure 3I**). mTEC C8 was a subpopulation of tuft-like cells highly expressing *Avil* and *Gnb3* (**Figures 3I** and **S4A**), as previously reported (33, 37). Notably, the mTEC C9 highly expressed CCR ligand chemokine *Ccl6*, *Ccl9*, and *Ccl20* (**Figures 3I** and **S4A**), reminiscent of the Gp2^+^ cells (35). Immunofluorescence staining identified Ccl6-expressing TECs mainly in medulla and the cortico-medullary junction (**Figure 3J**). Jennifer E. Cowan and colleagues demonstrated that Aire controls recirculation of peripheral Ccr6^+^ regulatory T cells (Treg) into the host thymus by regulating the Ccl20–Ccr6 axis (57). Thus, our data now pinpoint this interaction specifically to the Ccl6^+^ Ccl9^+^ Ccl20^+^ subset.

To reveal the relationships of mTEC subpopulations, we further did trajectory analysis to uncover the developmental path. Monocle analysis showed the path from pre-Aire mTECs to post-Aire mTECs through the intermediate proliferating cells (**Figure S4B**). Based on the trajectory analysis, mTEC C1 initiated the differentiation, through the proliferating state and emergence of Aire expression, and finally differentiated into mTEC C6, C8, and C9, which constituted the terminal state (**Figures S4B, C**). The scRNA-seq data combined with *in situ* staining together revealed the temporal dynamics of mTECs during organogenesis. The proliferative TECs at early thymus development (E11.5–E13.5) initiated mTEC lineage differentiation by upregulation of *Krtdap*, *Emp2*, *Ccl21a*, *Clu*, and *Krt5*, and then expanded in number and differentiated into Aire-expressing mTECs. A population of proliferative Aire-expressing mTECs may act as intermediates that facilitated the expansion of Aire^+^ mTECs. Aire^+^ mTECs continued to mature into Aire^-^ terminal cell types, mainly including Spink5^+^ cells, tuft-like cells, and Ccl6^+^ cells.

### The Plet1^+^ Cells Are Heterogeneous Across Development

**Figure 4:**
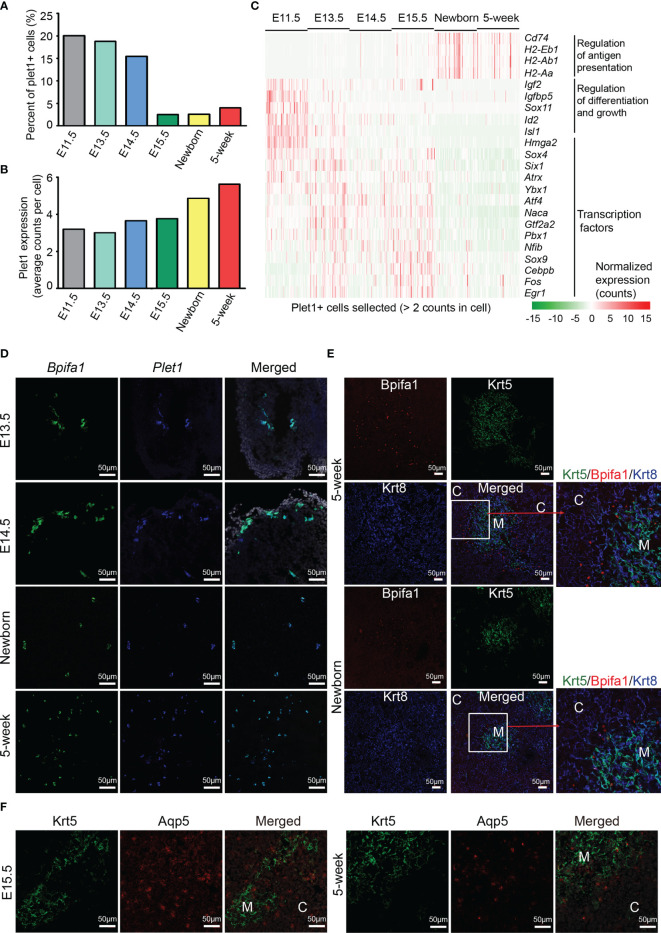
Molecular characteristics of Plet1^+^ cells during development. **(A)** Percentages of Plet1^+^ cells across the development from E11.5 to adult. Plet1^+^ cells were selected as ≧ 2 UMI counts in a single cell. **(B)** Average Plet1 expression (UMI counts) in the subpopulation of Plet1+ cells across the development. **(C)** Expression of differentially expressed genes in Plet1^+^ cells across development. (See also **Figure S5A** and **Table S4**) **(D)** Single-molecule RNA fluorescence *in situ* hybridization in thymus slides as indicated with probes targeting *Bpifa1* and *Plet1*. (See also **Figure S5B**) **(E)** Immunofluorescence staining of 5-week thymus slides with antibodies to Krt5 (green) and Bpifa1 (red). **(F)** Immunofluorescence staining of thymus slides at indicated stages with antibodies to Krt5 (green) and Aqp5 (red). All staining experiments were replicated at least 2 times, with 2 biological replicates for each experiment.

Identification of TEPCs has been partially defined by Plet1 expression (16, 19, 20). However, the characteristics of Plet1-positive cells in embryonic and adult thymus are still elusive. To this end, we selected the plet1-expressing cells (≧ 2 UMI counts in a single cell) from each developmental stage and reanalyzed their gene expression features. The relative abundance of *Plet1*-expressing cells dramatically decreased in proportion at E15.5 (**Figure 3B** and **Figure 4A**), while the level of *Plet1* expression was slightly enhanced in postnatal TECs (**Figure 4B**). We then defined expression signatures of the *Plet1*-expressing cells, highlighting the different gene expression profiling between embryonic and postnatal samples (**Figure S5A** and **Table S4**). Specifically, Plet1^+^ cells at E11.5 preferentially expressed genes that regulated cell differentiation and growth, including *Igf2*, *Igfbp5*, *Id2*, and *Isl1* (**Figure 4C**). Plet1^+^ cells at E13.5, E14.5, and E15.5 showed similar gene expression features, and upregulated a handful of transcription factors, including *Sox4*, *Sox9*, *Six1*, *Atrx*, *Ybx1*, *Naca*, *Gtf2a2*, *Pbx1*, *Atf4*, *Nfib*, *Cebpb*, *Fos*, and *Egr1* (**Figure 4C**). In contrast to the embryonic Plet1^+^ cells, postnatal Plet1^+^ cells (newborn and 5 weeks) highly expressed genes involved in regulation of antigen presentation, for example, *Cd74* and MHC molecules H2-Eb1, H2-Aa, and H2-Ab1 (**Figure 4C**). Thus, the transcriptional differences between embryonic Plet1^+^ cells and postnatal Plet1^+^ cells may reflect the decreased progenitor capacity in adult (58).

As we have noticed that mTEC C7 (**Figures 3A, I**) expressed both *Bpifa1* and *Plet1*, we further analyzed their expression in each developmental stage (**Figure S5B**) and in selected Plet1^+^ cells (**Figure S5C**). These results indicated that *Bpifa1* and *Plet1* may be co-expressed in a small population of mTECs. Using single-molecule RNA fluorescence *in situ* hybridization, we detected Bpifa1^+^ Plet1^+^ cells clustered in embryonic thymus sections, while dispersed in adult thymus sections (**Figure 4D**). Immunofluorescence staining identified Bpifa1 expression mainly at the cortico-medullary junction in newborn and 5-week-old thymus (**Figure 4E**). Interestingly, although Plet1^+^ cells lacked the expression of embryonic stem cell markers such as *Sox2*, they actually expressed several tissue-resident adult stem cell markers, including membrane protein *Aqp5*, which was recently identified as a specific marker of mouse and human adult epithelial stem cells (59), intestinal stem cell marker gene *Slc12a2* (60), and *Krt15*, which defined progenitors in the hair follicle, esophageal epithelium, and small intestine (61–63) (**Figure S5D**). We confirmed Aqp5 expression in thymus sections. Aqp5 was expressed in a clustered pattern in both medulla and cortex at E15.5, but mainly at the cortico-medullary junction in adult thymus (**Figure 4F**). In addition, Plet1^+^ cells expressed estrogen-responsive gene *Agr2*, and Annexin A1 and A3 (*Anxa1*, *Anxa3*), which were key regulators of epithelial cell proliferation and migration (64, 65) (**Figure S5E**). Plet1^+^ cells also expressed unique feature genes including *Cldn3*, *Cldn6*, *Ly6a*, *Krt6a*, *Krt19*, and *Gprc5a* (**Figure S5F**). Cldn3 has been identified as a marker of mTEC progenitors specified for Aire^+^ mTECs (66–68). Thus, the Plet1^+^ cells in adult thymus may still be heterogeneous, consisting of bipotent progenitors for both mTECs and cTECs and lineage committed progenitors, which needs further investigation. Our study provided transcriptomic differences between embryonic and postnatal Plet1^+^ cells and added new markers for identification of these progenitor cells.

### The Characteristics of Tissue-Restricted Antigen Expression During mTEC Development

**Figure 5:**
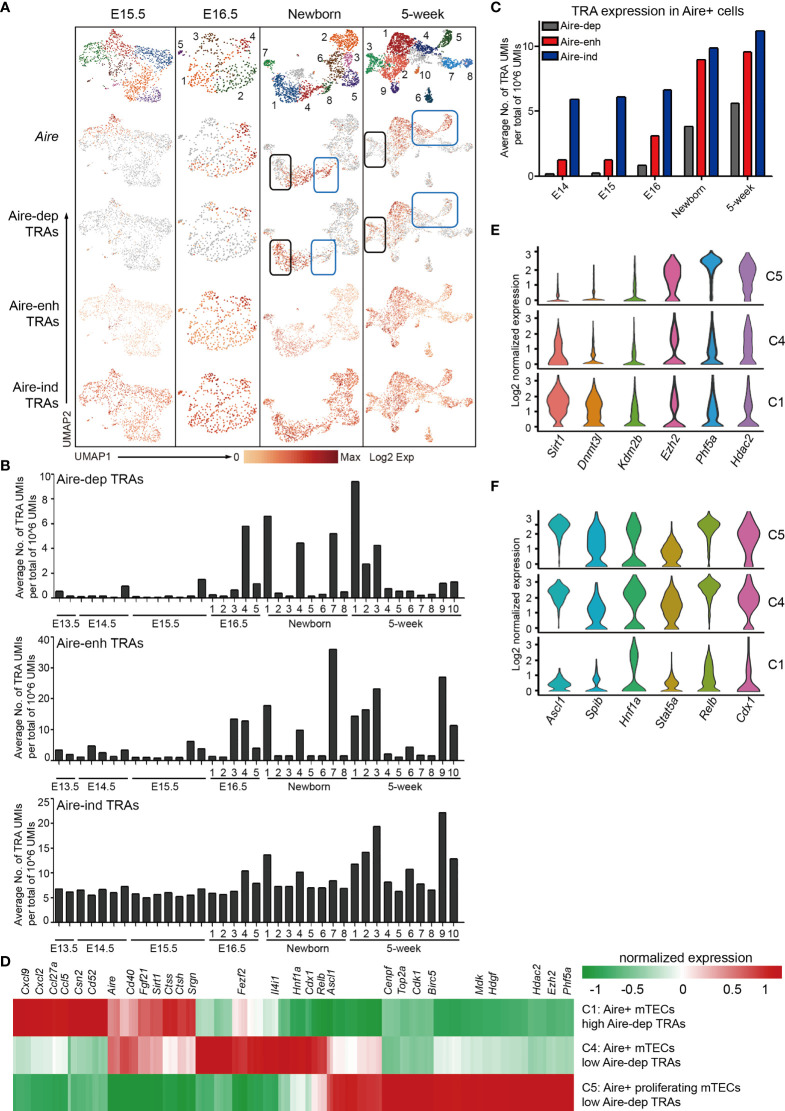
Characteristics of tissue-restricted antigen expression during mTEC development. **(A)** UMAP visualization of the expression of Aire, Aire-dep TRAs, Aire-enh TRAs, and Aire-ind TRAs in E15.5, newborn, and 5-week thymus. Each point corresponded to a single cell, and each color represented a cell cluster (first panel). Color represented maximum-normalized mean expression of Aire and TRAs (2–4 panels). **(B)** Average TRAs expression in each cell cluster in E13.5, E14.5, E15.5, E16.5, newborn, and 5-week thymus (upper panel, Aire-dep TRAs; middle panel, Aire-enh TRAs; lower panel, Aire-ind TRAs). **(C)** Average TRAs expression in Aire^+^ cells in E14.5, E15.5, E16.5, newborn, and 5-week thymus. Aire^+^ cells were selected as ≧ 2 UMI counts in a single cell. **(D)** Heat map showing differently expressed genes in C1 (cluster 1), C4, and C5 cells from 5-week thymus. **(E, F)** Violin plots showing the smoothened expression distribution of selected genes in C1, C4, and C5 cells from 5-week thymus.

The mTECs express an extensive library of tissue-restricted antigens (TRAs) termed promiscuous gene expression (PGE), which exhibits ordered co-expression (35). TRAs are categorized into 3 groups by their dependence on transcriptional regulation of Aire. Among these, transcription of 533 TRAs are entirely dependent on Aire (Aire-dependent, Aire-dep), 3,260 TRAs are enhanced by Aire (Aire-enhanced, Aire-enh), and transcription of 3,947 TRAs are independent of Aire (Aire-independent, Aire-ind) (69). We first analyzed the TRAs expression in scRNA-Seq data from E15.5, E16.5, newborn, and 5-week thymus because the mature mTECs were becoming evident until E15.5. Although the Aire expression was readily detected at E15.5, the expression of Aire-dep TRAs was still undetectable (**Figure 5A**). Similarly, in Newborn and 5-week data, expression of Aire-dep TRAs was also lacking in some clusters of Aire-expressing mTECs (**Figure 5A**, blue box). These results might be explained because these clusters just started to express Aire and the TRAs were thus not induced. In contrast, cells in Newborn C1 and 5-week C3 decreased the Aire expression, but still highly expressed Aire-dep TRAs (**Figure 5A**, black box). This was consistent with a recent report that TRA expression peaked as Aire expression decreased, implying Aire expression must be established before TRA expression can occur (44). The frequency of Aire^-/lo^ TRAs^+^ cells within total mTECs was about 12% in newborn thymus and 16% in 5-week thymus in our scRNA-seq data. Expression of Aire-enh TRAs was enhanced in Aire-expressing mTECs, and expression of Aire-ind TRAs was unchanged between Aire-expressing and Aire-negative subpopulations (**Figure 5A**).

To determine the TRAs expression more precisely, we analyzed the TRAs expression in each cluster at each developmental stage. The average expression frequencies of Aire-dep TRAs were significantly increased in E16.5 C4 (Cluster 4, Aire^+^ Csn2^+^ cells), Newborn C1 (Aire^+^ Csn2^+^ cells), Newborn C4 (Aire^+^ Csn2^+^ cells), Newborn C7 (Spink5^+^ cells), 5-week C1 (Aire^+^ Csn2^+^ cells), and 5-week C3 (Spink5^+^ cells) (**Figure 5B**, upper panel). Although Aire was significantly upregulated in clusters Newborn C8, 5-week C4, and 5-week C5, expression frequencies of Aire-dep TRAs were not upregulated (**Figure 5B**, blue box). Consistently, Aire-enh TRAs showed similar expression patterns with Aire-dep TRAs, except for the high expression of Aire-enh TRAs in 5-week C9 (Ccl6^+^ cells) (**Figure 5B**, middle panel). Expression frequencies of Aire-ind TRAs were similar across development, except for an increase in 5-week C3 (Spink5^+^ cells) and 5-week C9 (Ccl6^+^ cells) (**Figure 5B**, lower panel). Next, we selected the Aire^+^ cells (≧ 2 UMI counts in a single cell) and reanalyzed the expression frequencies of TRAs. Despite the high expression of Aire in all of these cells, the expression frequencies of TRAs were gradually increased along with the process of development (**Figure 5C**). Taken together, these results indicated that Aire expression must be established before Aire-dep TRAs expression, and other regulators may also be required to coordinate with Aire to promote their expression because expression of Aire-dep TRAs was still restrained in cells where Aire was already expressed.

To further identify the gene expression features underlying the mechanisms of different levels of Aire-dep TRAs expression, we compared the gene expression between clusters showing different Aire-dep TRAs expression patterns in 5-week thymus. 5-week C1 (highly expressed Aire and Aire-dep TRAs) was characterized by expression of chemokines *Cxcl9*, *Cxcl2*, *Ccl5*, and *Ccl27a*, genes potentially involved in protein processing such as *Ctss*, *Ctsh*, and *Srgn*, and protein deacetylase *Sirt1* (**Figure 5D**). 5-week C5 (highly expressed Aire but not Aire-dep TRAs) represented a subpopulation of mTECs that highly express cell cycle genes *Cenpf*, *Top2a*, *Cdk1*, and *Birc5*, growth-promoting factors *Mdk* and *Hdgf*, and epigenetic regulators *Ezh2*, *Phf5a*, and *Hdac2* (**Figures 5D, E**). Expression of transcription factors including *Ascl1*, *Relb*, *Cdx1*, *Hnf1a*, and *Fezf2*, which were highly expressed in 5-week C4 and C5, however, was decreased in 5-week C1 (**Figures 5D, F**).

### The Dynamic Heterogeneity of cTEC Across Development

**Figure 6:**
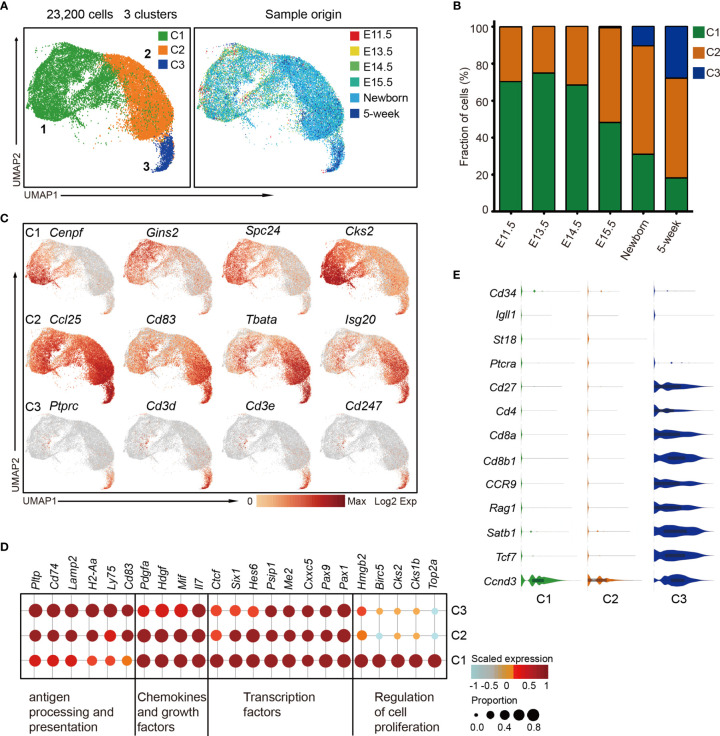
The temporal dynamics of cTEC diversity during development. **(A)** UMAP visualization of 23,200 cTECs with 3 subclusters. **(B)** Percentages of 3 cTEC subtypes across the development. **(C)** UMAP visualization of the expression of curated feature genes specific for 3 cTEC subtypes. **(D)** Dot plot for expression of marker genes in 3 major cTEC subtypes. Color represented maximum-normalized mean expression of marker genes in each cell group, and size indicated the proportion of cells expressing marker genes. (See also **Figure S6A**.) **(E)** Violin plots showing the smoothened expression distribution of selected genes specific for T-cell differentiation in cTEC subtypes.

cTECs play a vital role in early T-cell differentiation; however, the characterization of cTEC heterogeneity is still neglected by far. To investigate the cTEC differentiation trajectory, we therefore reanalyzed the cTEC population (as defined in **Figure 1B**) and projected them using UMAP analysis. Unsupervised clustering of cTECs revealed 3 major subclusters (cTEC C1, C2, and C3) with distinct gene expression features (**Figures 6A** and **S6A**), providing a greater richness of cell states than previously appreciated (33, 34, 44). Generally, all the cTECs expressed the well-established markers *Psmb11* and *Ccl25* (33). We observed the progressive decrease of cTEC C1 cells and increase of cTEC C2 cells in proportion during development (**Figure 6B**). While none of the cTEC C3 cells were present before E15.5, they were increasingly accumulated in postnatal thymus (**Figures 6B** and **S6B**).

cTEC C1 cells were characterized by high expression of cell proliferation-associated genes such as *Cenpf*, *Gins2*, *Spc24*, and *Cks2* (**Figure 6C**, upper panel). The data suggested that these cells might be highly proliferative, reminiscent of the perinatal cTECs in a recent study (27). cTEC C2 and C3 cells highly expressed a set of canonical markers of mature cTEC, including *Ccl25*, *Cd83*, *Tbata*, and *Isg20* (**Figure 6C**, middle panel), consistent with previous reports (27, 33). Notably, the cTEC C3 population also expressed genes *Ptprc*, *Cd3d*, *Cd3e*, and *Cd247* that were specific to T lymphocytes (**Figure 6C**, lower panel). This specific gene expression pattern reminded us of the thymic nurse cells (TNC) that were specialized cortical thymic epithelial cells enveloping the developing T cells within their intracellular vesicles (70). Thus, the TNC cell with one or more T cells inside was possibly captured as a single cell in our experiment system (see *Materials and Methods*). Transcriptomic profiling analysis revealed the commonly high expression of transcription factors *Pax1*, *Pax9*, *Cxxc5*, *Me2*, and *Psip1*; chemokines *Il7* and *Mif*; and growth factors *Hdgf* and *Pdgfa* in all cTECs (**Figure 6D**). cTEC C2 and C3 upregulated genes that were involved in antigen presentation and processing, including *Pltp*, *Cd74*, *Lamp2*, *H2-Aa*, *Ly75*, and *Cd83* (**Figure 6D**). We further analyzed the gene expression features that associated with T-cell differentiation. The TNC-enveloped T lymphocytes did not express *Cd34*, *Igll1*, *St18*, and *Ptcra* (**Figure 6E**), which were markers for early double negative T cells (36). However, they expressed cell surface receptors *Cd27*, *Cd4*, *Cd8a*, *Cd8b1*, and *Ccr9*; VDJ recombination gene *Rag1*; transcriptional regulators *Satb1* and *Tcf7*; and cyclin protein *Ccnd3* (**Figure 6E**), representing double-positive T-cell signatures (36).

Collectively, our data revealed 3 major cTEC states during development, including the immature, proliferative cTECs (cTEC C1), which dominated in embryonic thymus, mature cTECs (cTEC C2) which were equipped for antigen processing and presentation, and TNCs (cTEC C3) enveloping the double-positive T cells. Based on our data, cTECs showed less heterogeneity on the transcriptomic level compared with mTECs.

### The Dynamic Heterogeneity of Thymic Endothelial Cells Across Development

**Figure 7:**
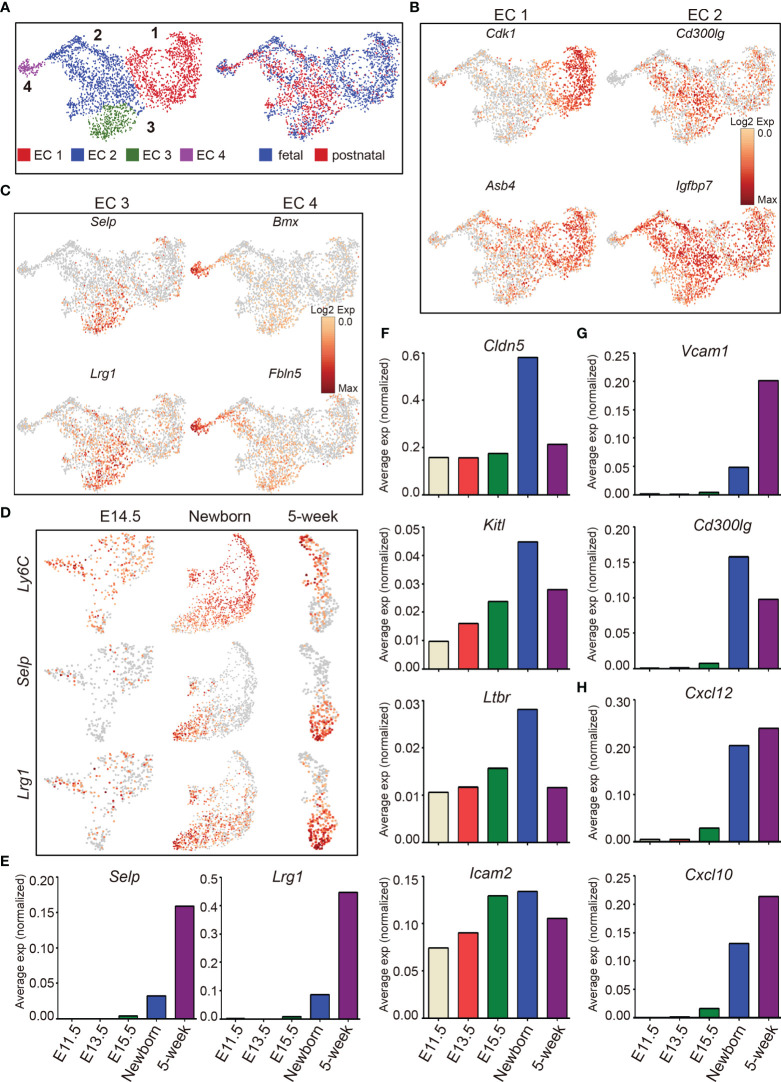
The dynamic heterogeneity of thymic endothelial cells across development. **(A)** UMAP visualization of the thymic endothelial cells colored by cell type, left panel and by sample origin, right panel (sample origin: fetal including E11.5, E12.5, E13.5, E14.5, E15.5, and E16.5; postnatal including newborn and 5-week thymus). **(B. C)** UMAP visualization of the expression of curated feature genes specific for 4 EC subtypes. **(D)** UMAP visualization of the expression of *Ly6C*, *Selp*, and *Lrg1* in E14.5, newborn, and 5-week samples. **(E–H)** Average expression (normalized counts) of selected genes in E11.5, E13.5, E15.5, newborn, and 5-week thymic ECs.

T-cell development depends on the continuous thymic homing of hematopoietic progenitor cells (HPCs) derived from the bone marrow. Thymic endothelial cells, especially those located within the perivascular spaces at the cortico-medullary junction area, play critical roles in thymic homing of HPCs (3, 71, 72). Unsupervised clustering of thymic endothelial cells revealed 4 major subclusters (EC 1, 2, 3, and 4) (**Figure 7A**, left panel), with fetal thymic endothelial cells enriched in cluster EC 1, and postnatal ECs enriched in cluster EC 2 and 3 (**Figure 7A**, right panel). Fetal ECs (EC 1) were characterized by high expression of *Cdk1* (a key factor in control of the eukaryotic cell cycle) and *Asb4*, a component of E3 ubiquitin-protein ligase complex that promotes differentiation of vascular lineage cells in an oxygen-dependent manner (73) (**Figure 7B**). Cluster EC 2 expressed higher *Cd300lg*, a receptor that mediates L-selectin-dependent lymphocyte rollings (74), and *Igfbp7* (**Figure 7B**). Cluster EC 3 was characterized by high expression of P-selectin (*Selp*) and *Lrg1* (**Figure 7C**). The gene expression specificity identified these cells as thymic portal ECs (TPECs) that mediate thymic progenitor cell entry (75, 76). Cluster EC 4 specifically expressed *Bmx* and *Fbln5* (**Figure 7C**). Bone marrow kinase in the X chromosome (*Bmx*) belongs to the protein tyrosine kinase family and is highly expressed in the endothelium of large arteries, starting between embryonic days 10.5 and 12.5 (77). *Bmx* can be upregulated in blood capillaries and Lyve1^+^ lymphatic vessels during endothelial remodeling (78), indicating that these cells may contribute to the generation of thymic vascular vessels. *Fbln5* was also reported to play important roles in vascular differentiation and maintenance of vascular integrity (79–81).

Ly6C^−^Selp^+^ thymic portal ECs (TPECs), of which the differentiation is controlled by Ltβr signaling, mediate thymic progenitor cell entry (75, 76). We therefore analyzed the dynamic expression of these genes during development. *Ly6C* was expressed as early as at E14.5 and continued to be expressed in adult (**Figure 7D**). In contrast, *Selp* was not expressed until after birth, and expression of *Ly6C* was reduced in cells expressing *Selp* (**Figure 7D**). Furthermore, we identified that *Lrg1* was specifically expressed in Ly6C^−^Selp^+^ thymic portal ECs (**Figures 7D, E**). Lrg1 was known to mediate TGF-beta-induced angiogenesis (82). Thus, the thymic entry of HPCs may coordinate the thymic vascular angiogenesis during thymus development.

Multiple factors from endothelial cells affect thymic homing of HPCs and egress of mature thymocytes including Selp (83), Ltβr (75, 84), membrane-bound form of Kitl (85, 86), Cldn5 (87), Cd300lg (88), and adhesion molecules Vcam and Icam (72, 89). Among these factors, *Selp* was upregulated in Ly6C^−^Selp^+^ thymic portal ECs in postnatal thymus (**Figures 7D, E**). *Cldn5*, *Kitl*, *Ltbr*, and *Icam2* began to express at E11.5 and peaked at birth (**Figure 7F**). *Vcam1* and *Cd300lg* were also upregulated in postnatal ECs (**Figure 7G**). Chemokines play important roles for thymic homing of HPCs and egress of mature thymocytes (90, 91). We found that *Cxcl10* and *Cxcl12* were highly expressed in postnatal thymic ECs (**Figure 7H**). These results indicated that the postnatal thymic ECs were equipped to mediate thymic homing and egress by upregulation of factors involved in cell–cell adhesion and chemotaxis.

### Coordinated Development of Thymic Stroma and Mesenchyme

We observed temporal changes in TEC populations starting from proliferative immature TECs toward the molecularly heterogeneous mTECs and mature cTECs including TNCs. Moreover, mesenchymal fibroblasts also changed during development. Three major subpopulations of mesenchymal fibroblasts were identified, of which Mes C1 (cluster 1) was mainly enriched in embryonic thymus and highly expressed cell cycle associated genes such as *Top2a* and *Ccnb2* (**Figures S7A, B**). Mes C2 was characterized by expression of *Gdf10* and *Igf1*, which were also expressed by Mes C1 cells (**Figures S7A, C**). Mes C3 highly expressed *Ndufa4l2* and *Cox4i2*, two important enzymes involved in energy metabolism (**Figures S7A, D**).

**Figure 8:**
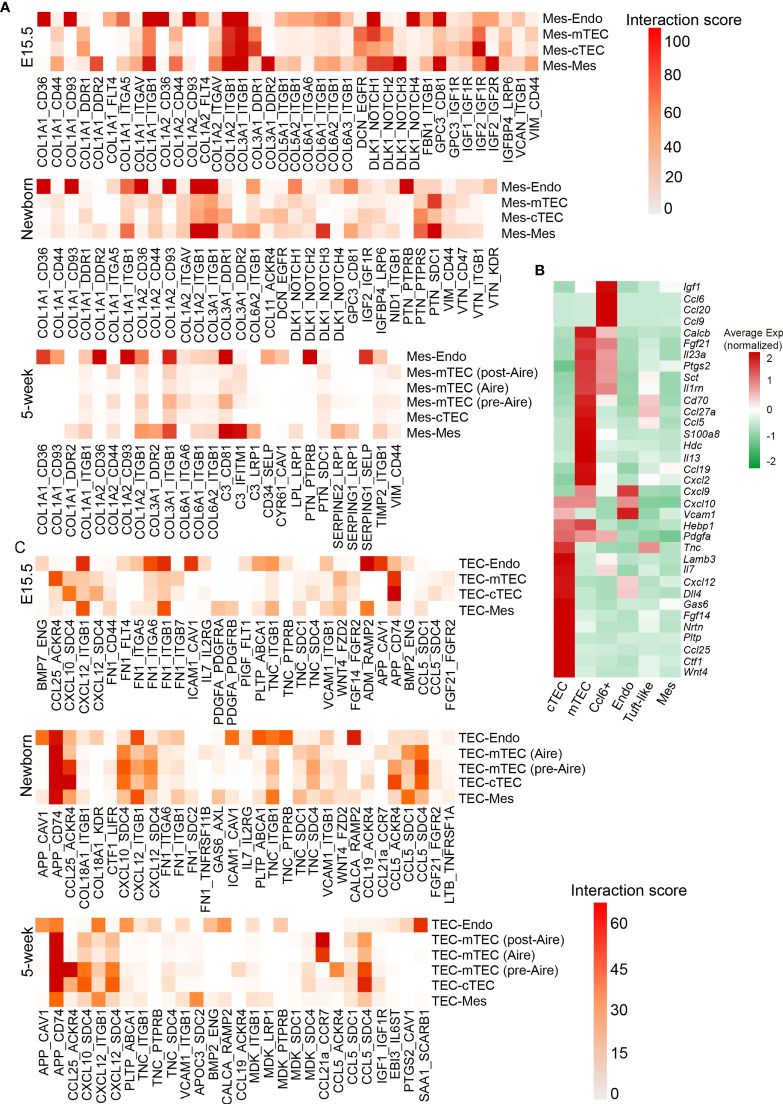
Quantification of ligand–receptor interactions occurring in the thymus. **(A)** Heat maps showing selected interaction scores calculated as the product of the average ligand expression of the first cell type (mesenchymal fibroblast) and average receptor expression of the second cell type. Cell-type labels were written as (cell type expressing the ligand) − (cell type expressing the receptor). (See also **Table S5**) **(B)** Heat maps showing ligand expression by cTEC, mTEC, Ccl6^+^ cells, endothelial cells (Endo), tuft-like cells, and mesenchymal fibroblast (Mes) in 5-week thymus. **(C)** Heat maps showing selected interaction scores calculated as the product of the average ligand expression of the first cell type (TEC) and average receptor expression of the second cell type. Cell-type labels were written as (cell type expressing the ligand) − (cell type expressing the receptor). (See also **Table S5**).

Mesenchymal fibroblast cells were demonstrated to be required for formation of thymic microenvironment by producing retinoic acid (92). Mesenchymal stromal cells or their extracellular vesicles showed great potential in promoting thymic epithelial cell expansion and differentiation (93, 94). To further investigate the factors mediating the development of TECs by mesenchymal fibroblast, we systematically investigated the ligand–receptor interactions specifically expressed across these cell types (see *Materials and Methods*). We used a reference list of known, literature-supported ligand–receptor pairs (47) and excluded the genes with no or rare expression in our data. Quantification of potential ligand–receptor interactions between all pairs of cell types based on gene expression revealed a handful of ligand–receptor pairs with high interaction scores (**Figure 8A** and **Table S5**). Collagens represented the most abundant ligand for a vast range of receptors including *Cd36*, *Cd93*, and *Itgb1*, which were highly expressed by endothelial cells (**Figure 8A**). Receptor tyrosine kinase signaling (Col3a1-Ddr1 and Igf2-Igf1r) and notch signaling (Dlk1-Notch1) in TECs were highlighted in fetal thymus but attenuated in adult (**Figures 8A** and **S7E**), which was consistent with much proliferative TECs in fetal stage.

As developing TECs also expressed dozens of ligands including chemokines, cytokines, and growth factors (**Figure 8B**), we then calculated the ligand–receptor interaction scores with ligands highly expressed on TECs. We observed several ligand–receptor pairs with potential interaction between TECs and endothelium, including App-Cav1, Cxcl12-Itgb1, Fn1-Itgb1, Icam1-Cav1, Adm-Ramp2, and Calca-Ramp2 mainly in fetal thymus (**Figure 8C** and **Table S5**). Thus, these interactions between TECs and endothelium may coordinate the formation of thymic vascular vessels. In addition, the autocrine interactions existed in cTECs that expressed both ligands and corresponding receptors, including Ccl25-Ackr4, Cxcl12-Sdc4, and Fgf14-Fgfr2. However, the strength of ligand–receptor interactions calculated based on the values of gene expression (see *Materials and Methods*) was markedly decreased in adult thymus (**Figures 8C** and **S7F**). mTECs specifically expressed *Ccl5* and *App*, and its potential receptors *Sdc4* and *Cd74* were expressed mainly on cTECs, indicating that potential signaling communications may exist between mTEC and cTEC through these ligand–receptor pairs (**Figures 8C** and **S7F**).

**Figure 9:**
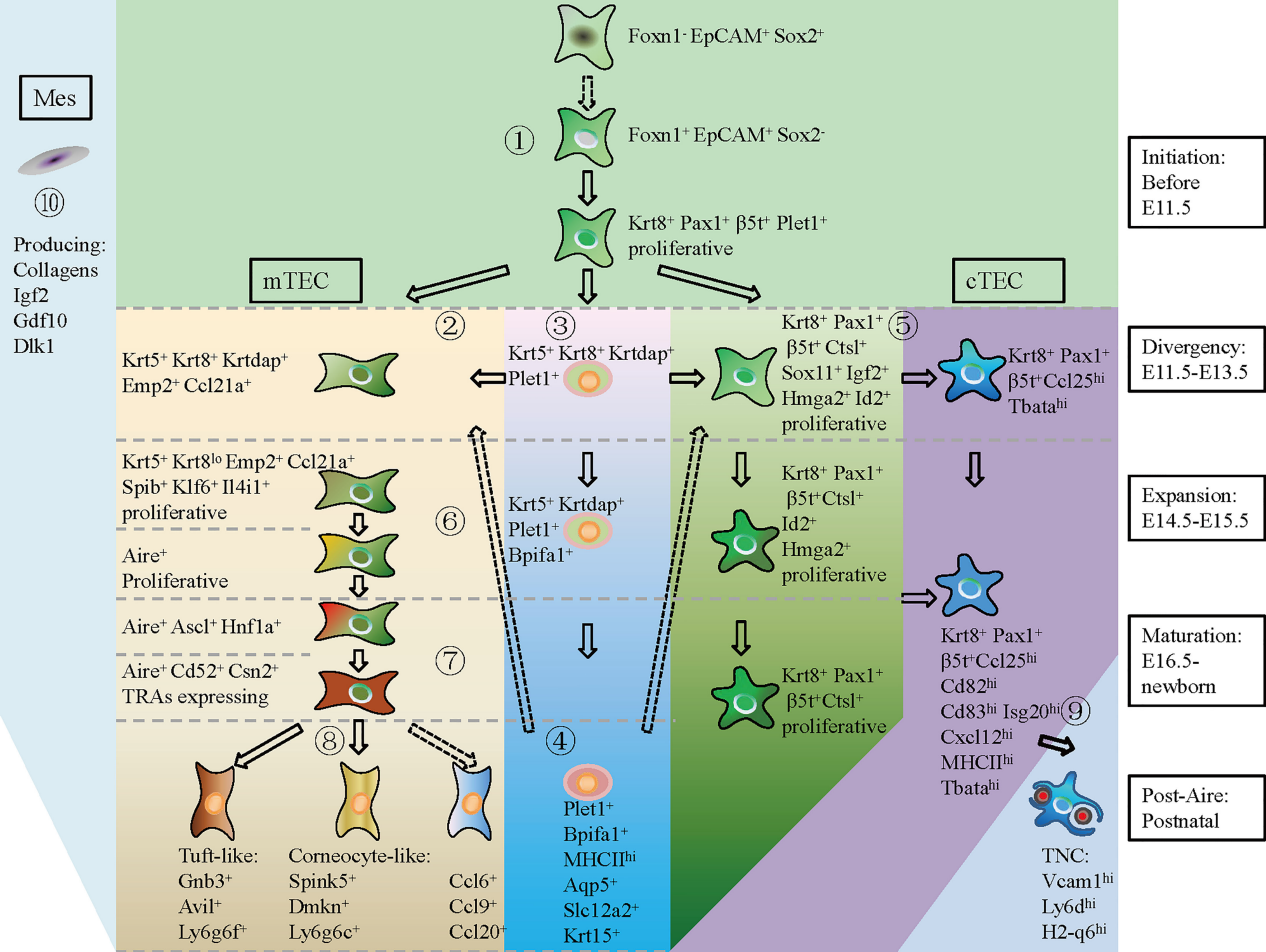
A hierarchical model of thymic epithelial cell differentiation with major lineage branches and feature genes during thymus organogenesis. At E11.5 and before (TEC initiation), Foxn1^-^ Epcam^+^ Sox2^+^ cells shut down Sox2 transcription and start expression of *Foxn1*, *Krt8*, *Pax1*, *β5t* ^+^ (*Psmb11*), and *Plet1* to establish the TEC identity (①). During E11.5–E13.5 (Divergency), a small population of TECs upregulated *Krt5*, *Krtdap*, *Emp2*, and *Ccl21a*, representing the mTEC progenitors (②). Within the Krt5^+^ cells, UMAP clustering analysis distinguished a subpopulation of Krt5^+^ Krt8^+^ Krtdap^+^ Plet1^+^ cells that expressed *Bpifa1*, *Aqp5*, *Slc12a2*, and *Krt15* in later development (③, ④). The Plet1^+^ cells at E11.5–E13.5 were previously demonstrated to be TEPCs, which gave rise to both mTEC and cTEC. However, the differentiation potential of Plet1^+^ cells in postnatal thymus needs to be further verified (④, dotted arrow). At this “Divergency” stage, most of the Krt8^+^ Pax1^+^ β5t^+^ TECs were highly proliferative and proceeded to differentiate into Ccl25^hi^ Tbata^hi^ cTECs (⑤). At E14.5–E15.5 (Expansion), proliferative mTEC progenitors decreased cTEC-specific gene expression, upregulated *Spib*, *Klf6*, and *Il4i1*, and started to express *Aire* (⑥). At E16.5-Newborn (Maturation), Aire^+^ mTECs highly expressed *Cd52*, *Csn2*, and Aire-dep TRAs (⑦). At postnatal stage (Post-Aire), mTECs continued to differentiate into tuft-like cells, corneocyte-like cells and Ccl6^+^ cells (⑧). Differentiation of Ccl6^+^ cells from Aire^+^ mTECs needs to be further demonstrated by *in vivo* lineage tracing evidence (dotted arrow). Maturation of cTEC in later development was characterized by upregulation of MHCII molecules and chemokines such as *Ccl25*, *Cxcl12*, and T cell-enveloping TNCs began to emerge at E16.5 (⑨). Mesenchymal fibroblast expressed kinds of ligands that provided molecular signaling to support TEC proliferation (⑩) (Mes, mesenchymal fibroblast; cTEC, cortical thymic epithelial cell; mTEC, medullary thymic epithelial cell). Solid arrow indicating a process has been experimentally validated, and dotted arrow indicating a process has not been validated.

In summary, based on the scRNA-seq data, we provided a hierarchical model of thymic epithelial cell differentiation including five consecutive stages: TEC lineage initiation, cortico-medullary thymic epithelial cell divergency, TEC expansion, TEC maturation, and post-Aire differentiation. This model highlighted the expression of common TEC genes and cTEC footprint genes when TEC lineage was initiated at E11.5. mTEC started differentiation by specific high expression of *Krt5*, *Krtdap*, *Emp2*, and *Ccl21a* and simultaneously downregulation of cTEC footprint genes at E11.5–E13.5. Maturation of mTEC was then achieved by expression of cell state-specific genes at corresponding stages of individual development. Maturation of cTEC was probably an intrinsic process in which cTECs upregulated expression of genes involved in antigen processing and presentation, and gradually downregulated expression of genes in control of cell proliferation. Embryonic mesenchymal fibroblasts may facilitate TEC proliferation by providing molecular signaling through Collogens, Igf2, Gdf10, and Dlk1 (**Figure 9**).

## Discussion

Using the scRNA-seq approach, we comprehensively analyzed the transcriptomic dynamics of thymic epithelial cells from embryonic to adult stages of thymus organogenesis. Droplet-based capturing of enriched non-hematopoietic cells and unsupervised clustering analysis revealed previously underappreciated cell types (Foxn1^-^ Epcam^+^ Sox2^+^ cell subpopulation, Bpifa1^+^ cell subpopulation, and pre-Aire proliferating immature mTEC) and, importantly, the developmental dynamics of thymic epithelial cells. Our data provided the detailed transcriptional characteristics of TECs at each developmental stage and the expression of TRAs in an exclusive circle of subpopulations.

Although recent studies have shed light on the thymic composition by revealing functionally distinct subpopulations (33, 34, 36, 39, 40), the characteristics of early TECs were neglected. To this end, our scRNA-seq data of TECs at the initiation stage of thymus development revealed a population of Foxn1^-^ Epcam^+^ Foxa1^+^ Foxe1^+^ Sox2^+^ cells that were mainly detected at E11.5. Due to the very limited cell number in E12.5 compared with other samples, the Sox2^+^ cells at this stage may be underestimated. We further analyzed the normalized average expression in EpCAM^+^ cells, and the average expression of Sox2 is 1.046 in E11.5, 0.076 in E12.5, and 0.057 in E13.5. Computational developmental trajectory analysis indicated that these cells preceded the Foxn1^+^ Epcam^+^ thymic epithelial cells. Sox2 expression has previously been identified in the third pharyngeal pouch from E9.5 to E10.5 (49). Thus, these data together indicated that the Foxn1^-^ Epcam^+^ Foxa1^+^ Foxe1^+^ Sox2^+^ cells might be upstream of Foxn1^+^ Epcam^+^ TECs. However, the differentiation of these Foxn1^-^ Epcam^+^ Foxa1^+^ Foxe1^+^ Sox2^+^ cells needs further investigation to confirm if they are actually upstream of Foxn1^+^ Epcam^+^ TECs or an alternative lineage of TECs independent of Foxn1^+^ TECs. The generation and rapid expansion of Foxn1^+^ Epcam^+^ Psmb11^+^ TECs (TEC 1) represented a population of biopotent progenitor cells, which diverged to give rise to Psmb11^hi^ Ccl25^hi^ cTECs (TEC 2) and Krt5^+^ Krtdap^+^ Emp2^+^ immature mTECs (TEC 3). These results were finely consistent with an *in vivo* fate-mapping study that both mTECs and cTECs were derived from progenitors that ever transcribed *Psmb11* during early embryogenesis (95). A small population of TECs at E11.5–E13.5 upregulated the expression of keratinocyte-specific genes *Krt5*, *Emp2*, and *Krtdap*; transcription factors *Irf6*, *Atf3*, *klf5*, *Spib*, and *Sox9*; metabolic genes *Plb1* and *Il4i1*; and chemokine *Ccl21a*, thus endowing themselves with mTEC potentials.

The expression of Ccl21a has long been identified in thymic epithelial cells (96, 97). Lucas Onder and colleagues reported that Pdpn^+^ TECs at the cortico-medullary junction represented a lineage committed progenitor for mTECs (53). These Pdpn^+^ progenitor cells were featured by high expression of Ccl21a (53). The lymphotoxin β receptor (LTβR) signaling regulated the expression of Ccl21a in mTECs (98), and deficiency of LTβR in mice significantly decreased the Ccl21^+^ Aire^−^ mTEC population but not Ccl21^+^ Aire^+^ mTEC and Ccl21^−^ Aire^+^ mTEC (99). To illustrate the cellular relationships in the branch of mTEC development, a study using Aire lineage tracing mice (Aire^CreERT2^; Rosa26^CAG-stopflox-zsGreen^) found that a proportion of Ccl21a^+^ cells expressed ZsGreen after tamoxifen treatment, indicating that Ccl21a^+^ cells have ever passed through an Aire-expressing state (44). This study suggested that Ccl21a-high population was not the progenitor of the Aire^+^ mTECs (44). However, a recent study using lineage tracing model β5t-Cre: Rosa26^flox-stop-flox-zsGreen^ revealed that the intertypical TECs (Ccl21a^+^ Krt5^+^) arising from aβ5t^+^ TEC progenitor population were lineage committed precursors for mature mTECs in adult (27). In our study covering TECs from embryonic thymus to adult, we revealed temporal dynamics of *Ccl21a* expression during thymus development (**Figure S3A**). *Ccl21a* was expressed as early as at E13.5 before Aire expression, and was ubiquitously expressed in all mTECs (including Aire^+^ mTEC and Aire^−^ mTEC) at embryonic stages. However, in adult thymus, only a small population of TECs sustained high expression of *Ccl21a*, while other mTEC subpopulations (including Aire^+^ mTEC and Aire^−^ mTEC) decreased *Ccl21a* expression. It is worth noting that *Ccl21a* expression was relatively decreased in Aire^+^ mTECs compared with Ccl21a-high population in adult thymus, but not unexpressed. It was consistent with the fact that part of the Ccl21a^+^ mTECs were Ccl21^+^ Aire^+^ mTECs in adult (44). Thus, our results indicated that *Ccl21a* was not only expressed before Aire expression in lineage committed progenitor cells but also expressed in Aire^+^ mTECs in both embryonic and adult thymus.

The immature mTEC further differentiated into a Aire^+^ proliferative intermediate at E14.5–E15.5. Maturation of mTEC first emerged at E16.5 as demonstrated by a subpopulation of Aire^+^ Cd52^+^ Csn2^+^ cells accompanied with expression of Aire-dependent TRAs. Post-Aire subclusters including Spink5^+^ Dmkn^+^ cells, tuft-like cells, and Ccl6^+^ Ccl20^+^ cells began to emerge at newborn and increased in number until adult. Spink5^+^ Dmkn^+^ cells and tuft-like cells were demonstrated to be developmentally derived from Aire^+^ cells by lineage tracing studies (33, 37, 100). We identified the Ccl6^+^ Ccl20^+^ cells within the Aire^+^ population in Newborn samples while they became a separate population and lost *Aire* expression in adult. These results indicated that the Ccl6^+^ Ccl20^+^ cells might be derived from Aire^+^ cells. The biological function and development of this cell type need further investigation.

ScRNA-seq of human thymus identified four specific cell types: (i) Epcam^+^ Myod1 ^+^ Myog1^+^ cells [TEC (myo)]; (ii) Epcam^+^ Neurod1^+^ Neurog1^+^ cells [TEC (neuro)], which resemble myoid cells and neuroendocrine cell respectively (36); (iii) myelin^+^ cells; and (iv) ciliated cells (39). We also identified a rare subpopulation of Epcam^+^ Myog1^+^ cells in newborn and adult mouse thymus, but Epcam^+^ Neurod1^+^ Neurog1^+^ cells, myelin^+^ cells, and ciliated cells were not detected (data not shown). These variations may be due to the species difference as ours and other studies regarding the heterogeneity of mouse TECs did not identify these cell types.

Plet1 has long been used to identify TEPCs in embryonic thymus (16, 19, 20), as well as in adult thymus (29). We selected the plet1-expressing cells (≧ 2 UMI counts in a single cell) from each developmental stage and reanalyzed their gene expression features. Plet1^+^ cells at early embryonic stages expressed genes that promote cell proliferation and common TEC specific transcription factors including *Sox4*, *Sox9*, *Six1*, *Pbx1*, *Atf4*, and *Fos*, while at postnatal stages, the Plet1^+^ cells highly expressed genes involved in regulation of antigen presentation, indicating that they have lost their primitive gene expression properties. However, the Plet1^+^ cells also expressed a set of genes that differed from other cell types. They expressed estrogen-responsive gene *Agr2*, which was involved in mammary epithelial proliferation and lobuloalveolar development (64). Annexin A1 and A3 (Anxa1, Anxa3), key regulators of epithelial cell migration (65), were highly expressed by the Plet1^+^ cells. Interestingly, although Plet1^+^ cells lacked the expression of embryonic stem cell markers, they actually expressed several tissue-resident adult stem cell markers, including membrane protein *Aqp5*, which was recently identified as a specific marker of mouse and human adult pyloric stem cells (59), intestinal stem cell marker gene *Slc12a2* (60), and *Krt15*, which defined progenitors in the hair follicle, esophageal epithelium, and small intestine (61–63). We also identified claudin genes *Cldn3*, which was expressed in lineage restricted mTEC progenitors, and *Cldn6* in the Plet1^+^ cells. Unsupervised clustering analysis of our scRNA-seq data revealed Plet1^+^ cells as a separate population at each developmental stage, indicating that these cells were specifically preserved during thymus organogenesis. As the potential of adult Plet1^+^ cells in regeneration of mTEC and cTEC is still controversial (28, 29, 95), the fate-mapping of adult Bpifa1^+^ Plet1^+^ cell differentiation may help to reveal the mechanisms of maintenance of thymic architecture in adult and regeneration of thymic tissue after injury or drug-induced thymic atrophy.

We further analyzed the TRAs expression in subclusters of each developmental stage. Generally, the expression levels of total TRAs (including Aire-dep, Aire-enh, and Aire-ind) were gradually increased along with development, concordant with the increased activities of T-cell development. Importantly, although Aire expression could be extensively detected at E14.5–E15.5 in thymus, the expression of Aire-dep TRAs was still kept silent in these Aire^+^ cells. Similarly, even in adult thymus, the expression of Aire-dep TRAs was not induced in certain Aire^+^ clusters, for instance, Aire^+^ proliferative mTECs. Aire-dep TRAs were highly expressed in Aire^+^ Csn2^+^ mTECs and Spink5^+^ Dmkn^+^ mTECs, in which *Aire* expression was becoming decreased. Thus, our results were concordant with the notion that *Aire* expression must be established before Aire-dep TRAs expression (39), and other regulators may also be required to coordinate with Aire to promote their expression. Aire^+^ proliferative mTECs (**Figure 5A**, 5-week-C5) and Aire^+^ low Aire-dep TRAs-expressing mTECs (**Figure 5A**, 5-week-C4) then represented different states preceding mTEC maturation.

The heterogeneity of cTECs during thymus organogenesis was largely neglected. All cTECs including the immature cTECs at early development (E11.5–E13.5) and mature cTECs in adult highly expressed feature genes such as *Pax1*, *Pax9*, and *Il7*. Proliferative cTECs (C1) highly expressed a set of cell cycle-associated genes, while mature cTECs (C2) and TNCs (C3) expressed much higher genes involved in antigen processing and presentation. We captured TNCs that were highlighted by expression of both cTEC and T-cell feature genes as one cTEC with inside enveloped T cell(s) was captured and sequenced as a single cell. However, the presence of TNCs revealed by scRNA-seq data was possibly underestimated due to the doublet removal in data processing. It is inevitable because current techniques in scRNA-seq data processing cannot discriminate natural cell aggregate and artificial doublet. This therefore led to a paradox because TNCs were multicellular complexes, and they were expected to be wiped out when removing the doublets. When processing our samples (E11.5, E12.5, E13.5, E14.5, E15.5, E16.5, newborn, and 5-week thymus) under the same condition individually, doublets from all samples were removed; however, TNCs were solely reserved in newborn and 5-week samples. This was consistent with the fact that TNCs sorted by magnetic CD45 depletion can only be detected from postnatal thymus (101). Thus, this subpopulation of EpCAM^+^ CD45^+^ cells were most likely TNCs rather than unremoved doublets.

Based on our scRNA-seq data and previous studies (95), lineage delineation algorithms predicted that early proliferative TECs with strong “cTEC footprint” at E11.5–E13.5 could give rise to both mTEC and cTEC. It is not sure if the proliferative cTECs (C1) at later developmental stages still possess the TEPC potentials. Unlike the mTEC differentiation model with phenotypically diverse cell types, the cTEC differentiation is simply achieved by downregulation of cell cycle-associated genes and upregulation of genes involved in antigen processing and presentation on the basis of our data.

We sampled early thymus (E11.5 and E12.5) from Foxn1-EGFP knockin mice in C57BL/6 background and late-stage thymus from wild-type C57BL/6 mice, thus resulting in an imperfect comparison between two strains. Comparison of our results to recent published data revealed similarities on feature gene expression and cell subpopulations, for example, immature cTEC population at E12.5 and E13.5 (34), early mTEC at E13.5 (34), mature mTEC and tuft-like cells (33), Ccl21a^+^ Pdpn^+^ TEC (27), and proliferating immature mTEC (44). Thus, these similar conclusions have been drawn, clearly supporting the methodology and data interpretation made upon this mixed dataset.

## Data Availability Statement

The datasets presented in this study can be found in online repositories. The names of the repository/repositories and accession number(s) can be found below: GEO, GSE192480.

## Ethics Statement

The animal study was reviewed and approved by Guangdong Medical University.

## Author Contributions

Conceptualization, PC Methodology, PC, HG, and MC. Investigation, HG, MC, KD, YY, JS, MN, CTX, WF, CO, DH, LZL, LXL, YL, HS, XC, JW, CLX, and XD. Writing—original draft, PC. Writing—Review & Editing, HG, LM, and MC. Supervision, PC. All authors contributed to the article and approved the submitted version.

## Funding

This work was supported by grants from Longhua District Science and Technology Innovation Fund (201803), Longhua District Key Laboratory of Genomics and Precision Medicine (20170913A0410026), Guangdong Basic and Applied Basic Research Foundation (2019A1515011009, 2021A1515010683, 2020A1515010225, and 2021A1515010955), Shenzhen Foundation of Science and Technology (JCYJ20180306172449376, JCYJ20180306172459580, and JCYJ20180306172502097), and Shenzhen Longhua District Foundation of Science and Technology (SZLHQJCYJ202002).

## Conflict of Interest

The authors declare that the research was conducted in the absence of any commercial or financial relationships that could be construed as a potential conflict of interest.

## Publisher’s Note

All claims expressed in this article are solely those of the authors and do not necessarily represent those of their affiliated organizations, or those of the publisher, the editors and the reviewers. Any product that may be evaluated in this article, or claim that may be made by its manufacturer, is not guaranteed or endorsed by the publisher.

## Supplementary Material

Supplementary Table 4 Non-scaled gene expression (UMI counts) for Plet1^+^ cells related to **Figure 4**.

## Abbreviations

cTEC, cortical thymic epithelial cell; mTEC, medullary thymic epithelial cell; RFTOC, reaggregate fetal thymus organ culture; scRNA-seq, single-cell RNA sequencing; TEC, thymic epithelial cell; TEPC, thymic epithelial progenitor cell.
