# Supplementary material for: The Lineage Differentiation and Dynamic Heterogeneity of Thymic Epithelial Cells During Thymus Organogenesis
Source: Front Immunol. 2022 Feb 22;13:805451. doi: 10.3389/fimmu.2022.805451 (PMC8901506; doi:10.3389/fimmu.2022.805451)
Supplement: Supplementary file 1 [file DataSheet_1.pdf]

SUPPLEMENTAL INFORMATION
SUPPLEMENTAL FIGURES

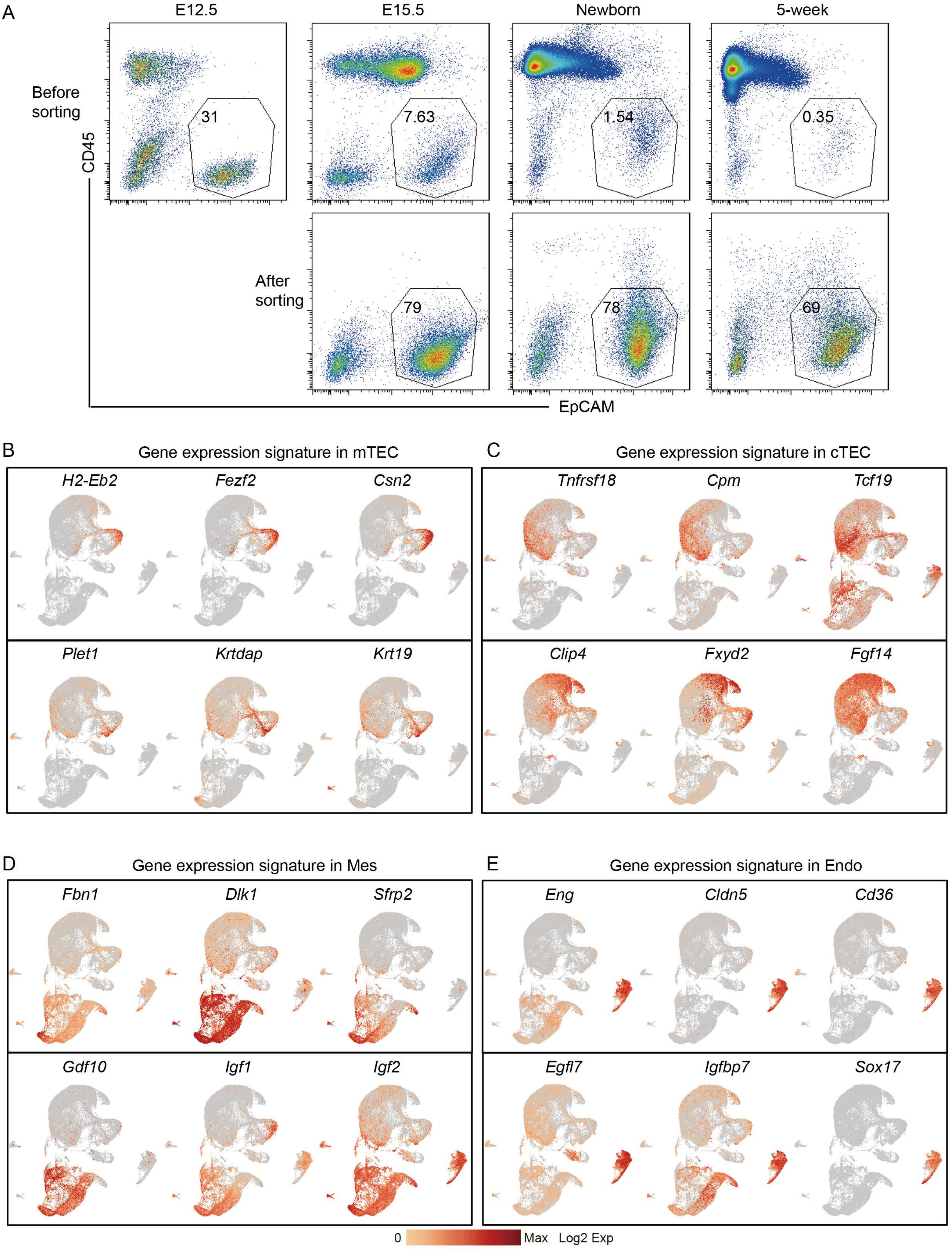

Figure. S1 Feature gene expression of the major cell types.

(A) Representative FACS analysis of $Cd45^-$ $EpCAM^+$ TECs before and after magnetic depletion of hematopoietic cells. (n=3).

(B-E) UMAP visualization of the expression of curated feature genes specific for **(B)** mTEC, **(C)** cTEC, **(D)** endothelial cell (Endo), **(E)** mesenchymal fibroblasts (Mes).

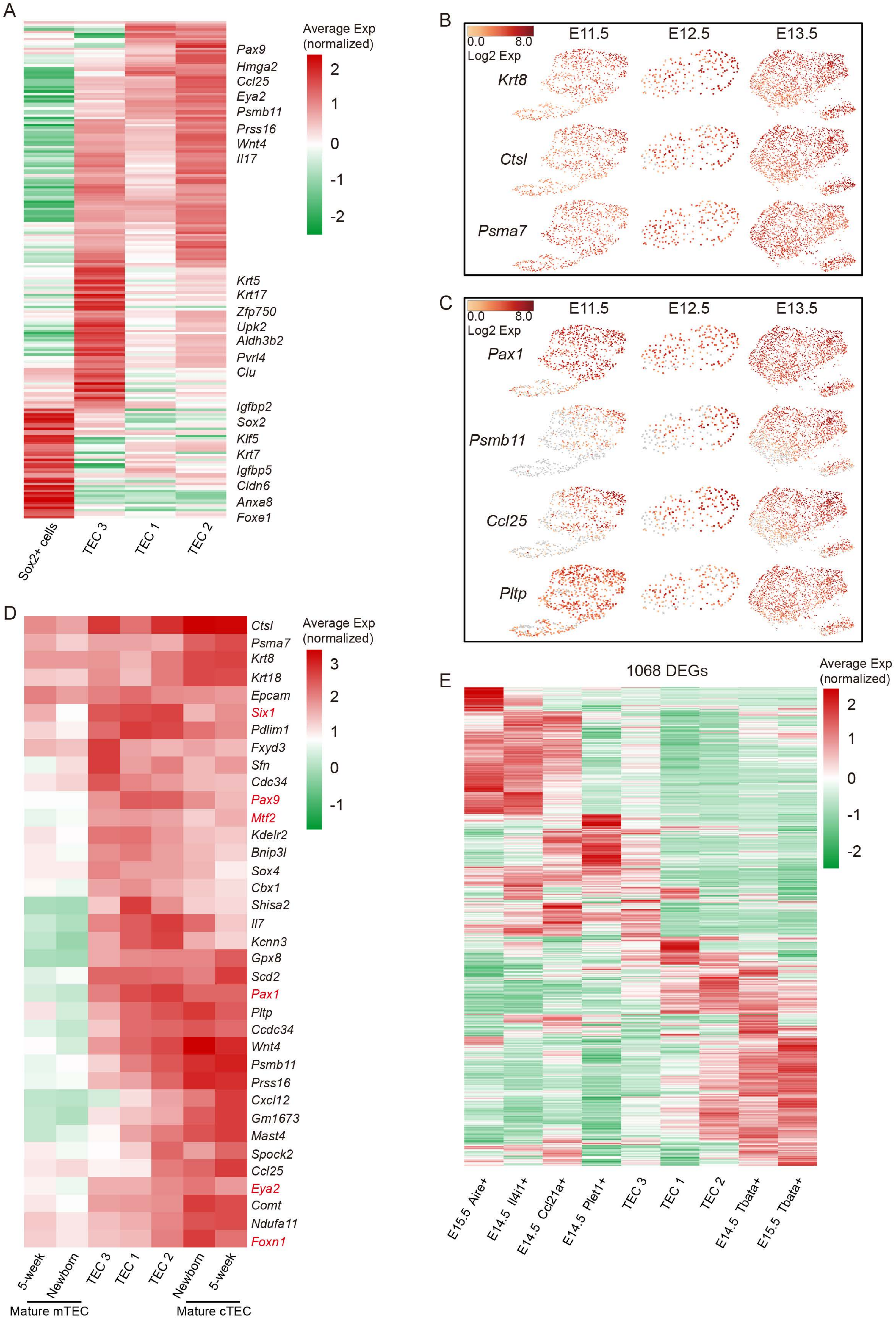

A
Average Exp (normalized)
2
1
0
-1
-2
Pax9
Hmga2
Ccl25
Eya2
Psmb11
Prss16
Wnt4
Il17
Krt5
Krt17
Zfp750
Upk2
Aldh3b2
Pvrl4
Clu
Igfbp2
Sox2
Klf5
Krt7
Igfbp5
Cldn6
Anxa8
Foxe1
Sox2+ cells
TEC 3
TEC 1
TEC 2
B
0.0
8.0
Log2 Exp
E11.5
E12.5
E13.5
Krt8
Ctsl
Psma7
C
0.0
8.0
Log2 Exp
E11.5
E12.5
E13.5
Pax1
Psmb11
Ccl25
Pltp
D
Average Exp (normalized)
3
2
1
0
-1
Ctsl
Psma7
Krt8
Krt18
Epcam
Six1
Pdlim1
Fxyd3
Sfn
Cdc34
Pax9
Mtf2
Kdelr2
Bnip3l
Sox4
Cbx1
Shisa2
Il7
Kcnn3
Gpx8
Scd2
Pax1
Pltp
Ccdc34
Wnt4
Psmb11
Prss16
Cxcl12
Gm1673
Mast4
Spock2
Ccl25
Eya2
Comt
Ndufa11
Foxn1
5-week
Newborn
TEC 3
TEC 1
TEC 2
Newborn
5-week
Mature mTEC
Mature cTEC
E
1068 DEGs
Average Exp (normalized)
2
1
0
-1
-2
E15.5 Aire+
E14.5 Il4i1+
E14.5 Ccl21a+
E14.5 Plet1+
TEC 3
TEC 1
TEC 2
E14.5 Tbata+
E15.5 Tbata+

Figure. S2 Gene expression profiling of the early TECs at E11.5-E13.5.

(A) Heat map showing differentially expressed genes in TEC 1, TEC 2, TEC 3 and $Sox2^{+}$ cells of early thymus (E11.5-E13.5). Gene expression levels were maximum-normalized and smoothed. Genes were grouped by their expression patterns.

(B-C) UMAP visualization of the expression of cTEC feature genes in early TECs.

(D) Heat map showing expression of mature cTEC-specific genes in TEC 1, TEC 2, TEC 3 from E11.5-E13.5, mature mTEC and mature cTEC from newborn and 5-week thymus. Genes were grouped by their expression patterns. Genes in red are transcription factors.

(E) Heat map showing 1,068 differentially expressed genes in clusters of TEC 1, TEC 2, TEC 3 from E11.5-E13.5 thymus, and mTEC, cTEC from E14.5, E15.5 thymus. Expression levels were maximum-normalized and smoothed. Genes were grouped by their expression patterns. (DEGs, differentially expressed genes).

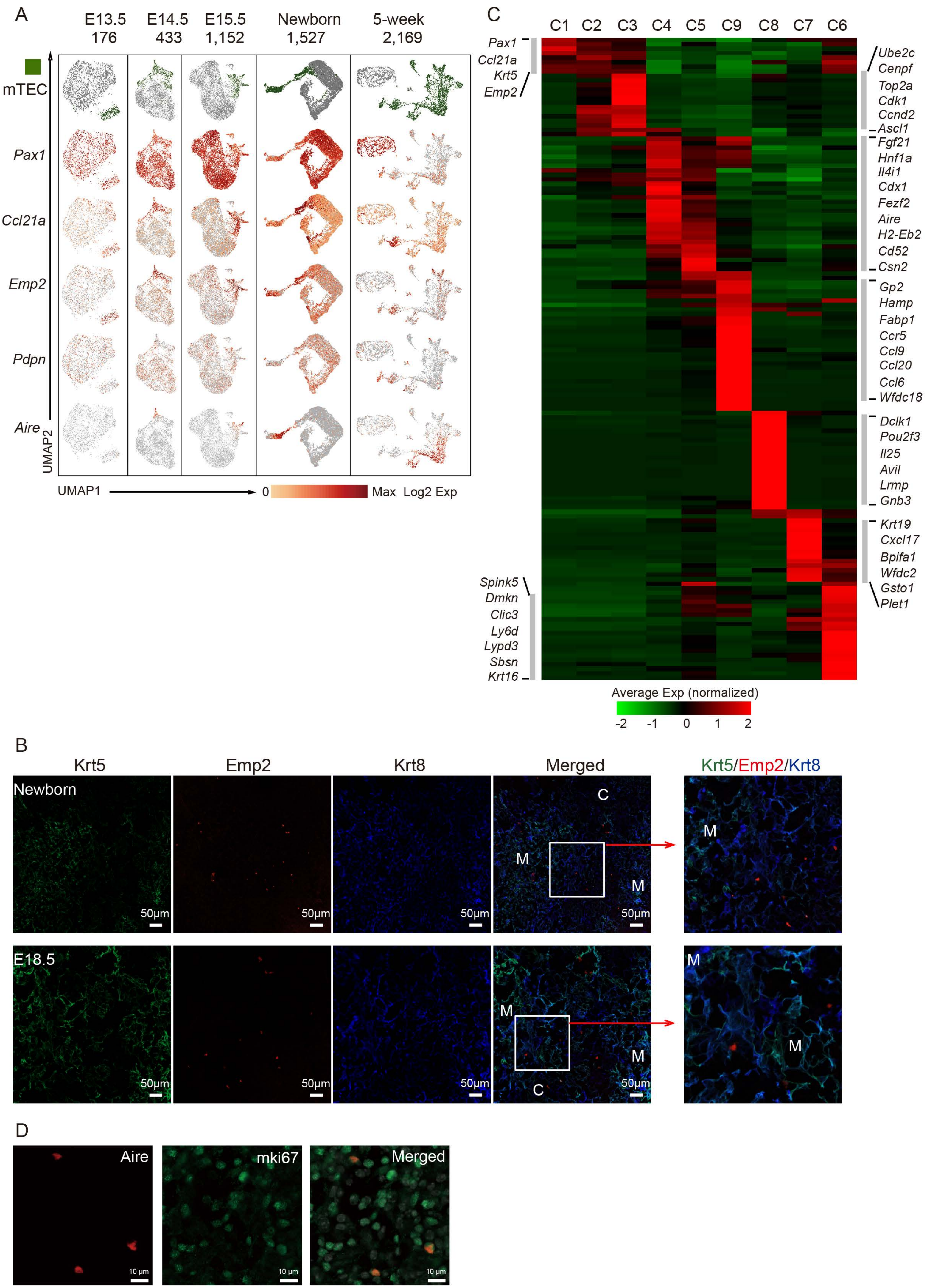

A
E13.5 176
E14.5 433
E15.5 1,152
Newborn 1,527
5-week 2,169
mTEC
Pax1
Ccl21a
Emp2
Pdpn
Aire
UMAP2
UMAP1
0
Max Log2 Exp
C
C1 C2 C3 C4 C5 C9 C8 C7 C6
Pax1
Ccl21a
Krt5
Emp2
Ube2c
Cenpf
Top2a
Cdk1
Ccnd2
Ascl1
Fgf21
Hnf1a
Il4i1
Cdx1
Fezf2
Aire
H2-Eb2
Cd52
Csn2
Gp2
Hamp
Fabp1
Ccr5
Ccl9
Ccl20
Ccl6
Wfdc18
Dclk1
Pou2f3
Il25
Avil
Lrmp
Gnb3
Krt19
Cxcl17
Bpifa1
Wfdc2
Gsto1
Plet1
Spink5
Dmkn
Clic3
Ly6d
Lypd3
Sbsn
Krt16
Average Exp (normalized)
-2 -1 0 1 2
B
Krt5
Emp2
Krt8
Merged
Krt5/Emp2/Krt8
Newborn
E18.5
C
M
50μm
D
Aire
mki67
Merged
10 μm

Figure. S3 The molecular characteristics of mTEC diversity during development.

(A) UMAP visualization of the expression of *Pax1*, *Ccl21a*, *Emp2*, *Pdpn*, and *Aire* in mTECs at each developmental stage. Numbers on the diagram indicated the number of mTECs in our data. Green points indicated subpopulations of mTECs.

(B) Immunofluorescence staining of newborn and E18.5 thymus slides with antibodies to Krt5 (green), Emp2 (red), and Krt8 (blue). All staining experiments were replicated at least 2 times, and 2 biological replicates for each experiment.

(C) Heat map showing differentially expressed genes in 9 clusters of mTECs. Genes were grouped by their expression patterns.

(D) Immunofluorescence staining of 5-week thymus slides with antibodies to mKi67 (green), Aire (red). All staining experiments were replicated at least 2 times, and 2 biological replicates for each experiment.

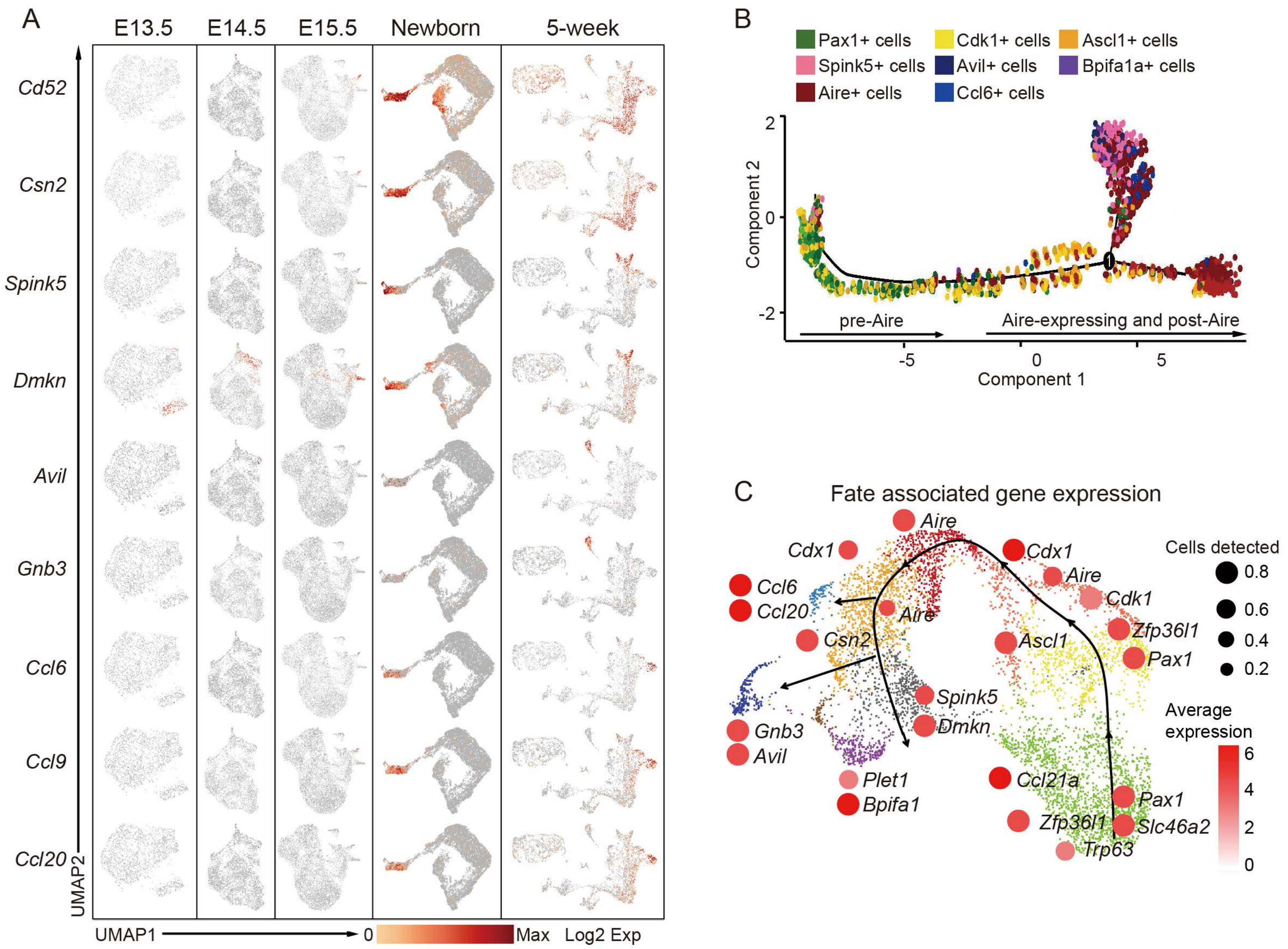

A
E13.5
E14.5
E15.5
Newborn
5-week
Cd52
Csn2
Spink5
Dmkn
Avil
Gnb3
Ccl6
Ccl9
Ccl20
UMAP2
UMAP1
0
Max
Log2 Exp
B
Pax1+ cells
Cdk1+ cells
Ascl1+ cells
Spink5+ cells
Avil+ cells
Bpifa1a+ cells
Aire+ cells
Ccl6+ cells
Component 2
Component 1
pre-Aire
Aire-expressing and post-Aire
C
Fate associated gene expression
Cells detected
Average expression
Aire
Cdx1
Ccl6
Ccl20
Csn2
Spink5
Dmkn
Gnb3
Avil
Plet1
Bpifa1
Cdk1
Zfp36l1
Pax1
Ascl1
Ccl21a
Slc46a2
Trp63

Figure. S4 Pseudotime trajectory analysis for mTECs.

(A) UMAP visualization of the expression of *Cd52*, *Csn2*, *Spink5*, *Dmkn*, *Avil*, *Gnb3*, *Ccl6*, *Ccl9*, and *Ccl20* in mTECs at each developmental stage.

(B) The ordering of mTECs from E13.5 to adult thymus along pseudotime in a two-dimensional state-space defined by Monocle2. Cell orders were inferred from the expression of most dispersed genes across mTECs. Each point corresponded to a single cell, and each color represented a cell cluster.

(C) Dot plot showing expression of fate associated genes in mTEC clusters. Color represented maximumnormalized mean expression of genes in each cell cluster, and size indicated the proportion of cells expressing marker genes.

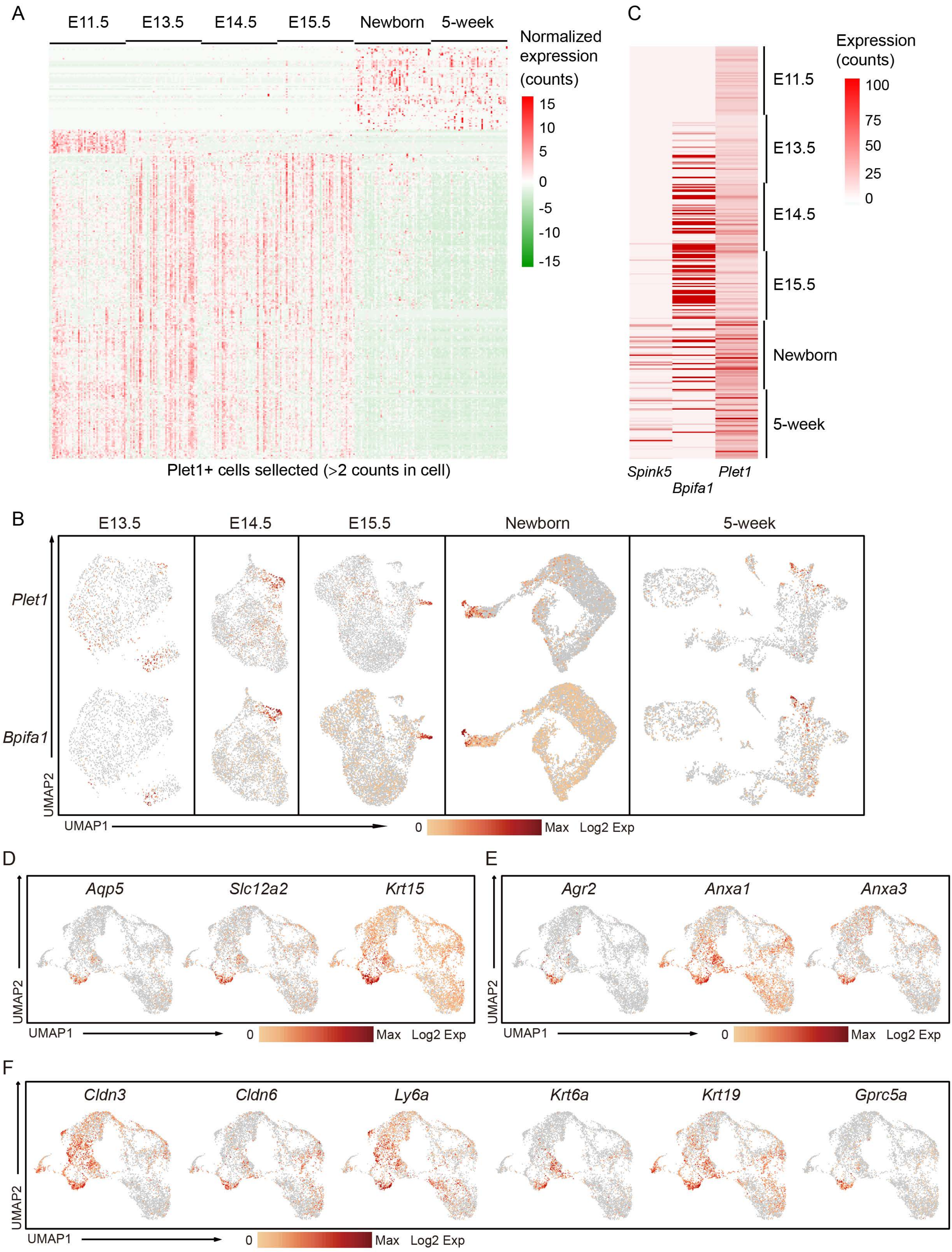
A
E11.5
E13.5
E14.5
E15.5
Newborn
5-week
Normalized expression (counts)
15
10
5
0
-5
-10
-15
Plet1+ cells sellected (>2 counts in cell)
C
Expression (counts)
100
75
50
25
0
E11.5
E13.5
E14.5
E15.5
Newborn
5-week
Spink5
Bpifa1
Plet1
B
E13.5
E14.5
E15.5
Newborn
5-week
Plet1
Bpifa1
UMAP2
UMAP1
0
Max
Log2 Exp
D
Aqp5
Slc12a2
Krt15
UMAP2
UMAP1
0
Max
Log2 Exp
E
Agr2
Anxa1
Anxa3
UMAP2
UMAP1
0
Max
Log2 Exp
F
Cldn3
Cldn6
Ly6a
Krt6a
Krt19
Gprc5a
UMAP2
UMAP1
0
Max
Log2 Exp

Figure. S5 Molecular characteristics of Plet1$^+$ cells during development.

(A) Clustering analysis of differentially expressed genes in Plet1$^+$ cells from 6 developmental stages.

(B) UMAP visualization of the expression of *Bpifa1* and *Plet1* in indidcated developmental stages.

(C) Expression of selected genes in Plet1$^+$ cells.

(D-F) UMAP visualization of the expression of indicated genes that specific to Bpifa$^+$ Plet1$^+$ cells.

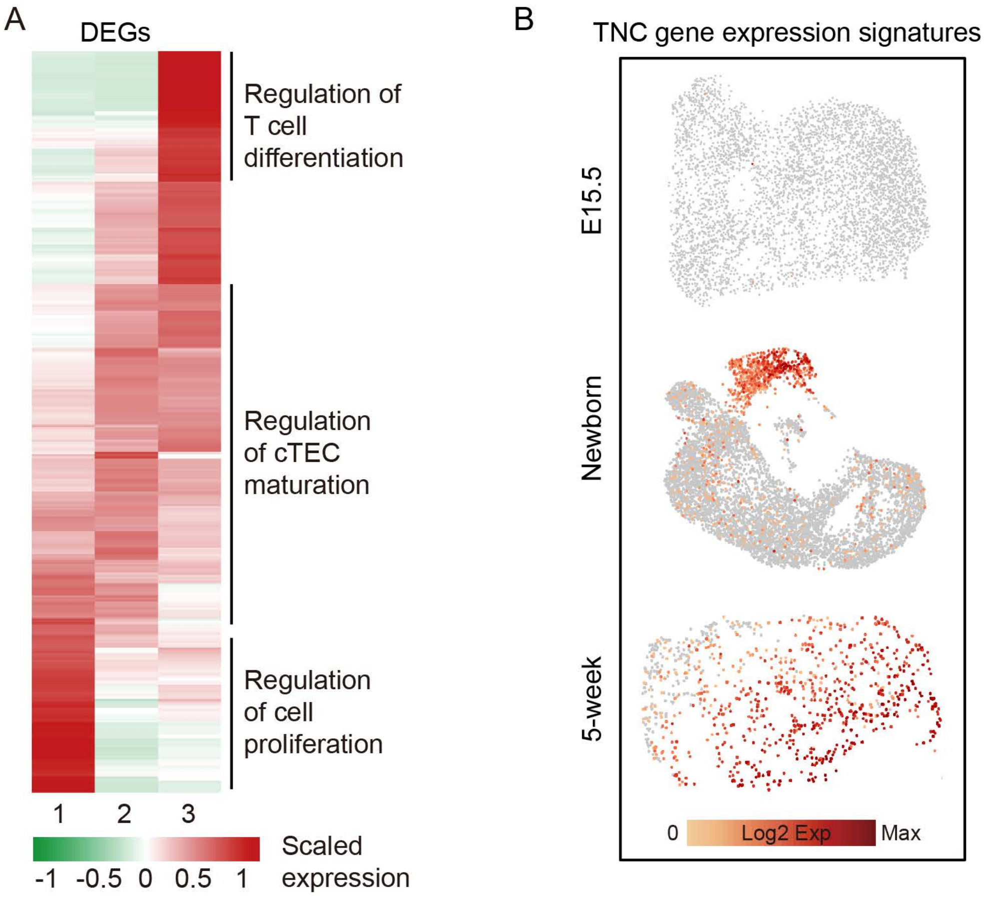

Figure. S6 Gene expression profiling of 3 subclusters of cTECs.

(A) Heat map showing differentially expressed genes in subtypes of cTECs. Genes were grouped by their functional categories and expression patterns. (DEGs, differentially expressed genes).

(B) UMAP visualization of the expression of the TNC feature genes in cTECs.

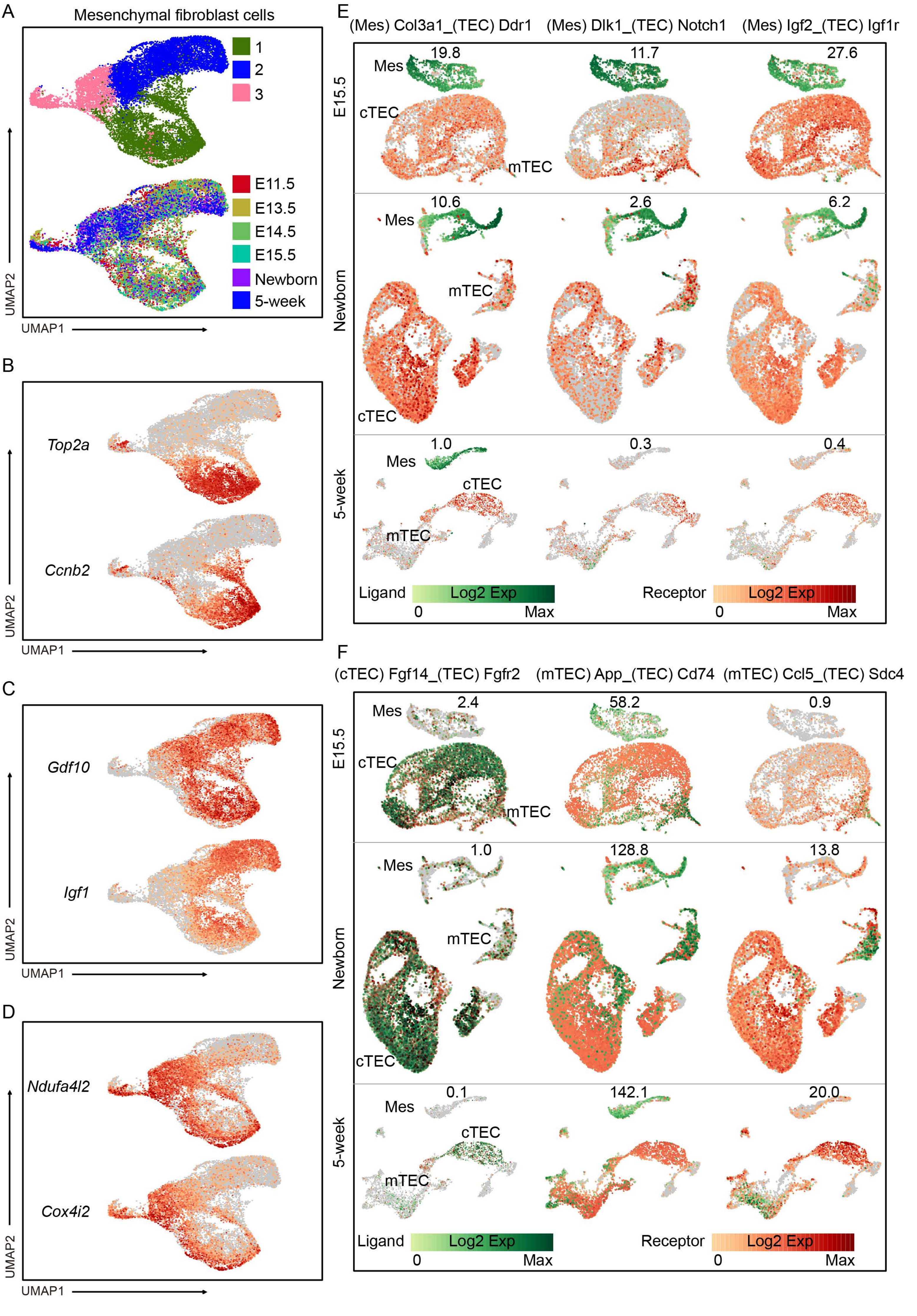
A
Mesenchymal fibroblast cells
1
2
3
E11.5
E13.5
E14.5
E15.5
Newborn
5-week
UMAP2
UMAP1
B
Top2a
Ccnb2
C
Gdf10
Igf1
D
Ndufa4l2
Cox4i2
E
(Mes) Col3a1_(TEC) Ddr1
(Mes) Dlk1_(TEC) Notch1
(Mes) Igf2_(TEC) Igf1r
E15.5
19.8
11.7
27.6
Mes
cTEC
mTEC
Newborn
10.6
2.6
6.2
5-week
1.0
0.3
0.4
Ligand
Log2 Exp
0
Max
Receptor
F
(cTEC) Fgf14_(TEC) Fgfr2
(mTEC) App_(TEC) Cd74
(mTEC) Ccl5_(TEC) Sdc4
2.4
58.2
0.9
1.0
128.8
13.8
0.1
142.1
20.0

Figure. S7 Ligand-receptor interactions between mesenchymal fibroblast and TECs.

(A) UMAP visualization of mesenchymal fibroblast cells, with each cell colorcoded for each subcluster (upper panel), and the sample origin (lower panel).

(B-D) UMAP visualization of the expression of **(B)** *Top2a*, *Ccnb2*, **(C)** *Gdf10*, *Igf1*, **(D)** *Ndufa4l2*, *Cox4i2*.

(E) UMAP visualization of the expression of ligand-receptor pairs (Col3a1_Ddr1, Dlk1_Notch1, Igf2_Igf1r), with each cell colorcoded for mesenchyme expressed ligands (green) and TEC expressed receptors (red).

(F) UMAP visualization of the expression of ligand-receptor pairs (Fgf14_Fgfr2, App_Cd74, Ccl5_Sdc4), with each cell colorcoded for TEC expressed ligands (green) and receptors (red). Numbers on the diagram were ligand-receptor interaction scores as indicated.
